# *CircARAP2* controls sMICA-induced NK cell desensitization by erasing CTCF/PRC2-induced suppression in early endosome marker *RAB5A*

**DOI:** 10.1007/s00018-024-05285-1

**Published:** 2024-07-24

**Authors:** Feifei Guo, Nawen Du, Xue Wen, Zhaozhi Li, Yantong Guo, Lei Zhou, Andrew R. Hoffman, Lingyu Li, Ji-Fan Hu, Jiuwei Cui

**Affiliations:** 1 Cancer Center, The First Hospital of Jilin University, 71 Xinmin Street, Changchun, 130021 China; 2 Stanford University School of Medicine, VA Palo Alto Health Care System, Palo Alto, CA 94304 USA

**Keywords:** sMICA, NK cell desensitization, circRNA, NKG2D, Endocytosis, Histone methylation

## Abstract

Natural killer cells (NK) are the "professional killer" of tumors and play a crucial role in anti-tumor immunotherapy. NK cell desensitization is a key mechanism of tumor immune escape. Dysregulated NKG2D-NKG2DL signaling is a primary driver of this desensitization process. However, the factors that regulate NK cell desensitization remain largely uncharacterized. Here, we present the first report that circular RNA *circARAP2* (hsa_circ_0069396) is involved in the soluble MICA (sMICA)-induced NKG2D endocytosis in the NK cell desensitization model. *CircARAP2* was upregulated during NK cell desensitization and the loss of *circARAP2* alleviated NKG2D endocytosis and NK cell desensitization. Using Chromatin isolation by RNA purification (ChIRP) and RNA pull-down approaches, we identified that *RAB5A*, a molecular marker of early endosomes, was its downstream target. Notably, transcription factor CTCF was an intermediate functional partner of *circARAP2*. Mechanistically, we discovered that *circARAP2* interacted with CTCF and inhibited the recruitment of CTCF-Polycomb Repressive Complex 2 (PRC2) to the promoter region of *RAB5A*, thereby erasing histone H3K27 and H3K9 methylation suppression to enhance *RAB5A* transcription. These data demonstrate that inhibition of *circARAP2* effectively alleviates sMICA-induced NKG2D endocytosis and NK cell desensitization, providing a novel target for therapeutic intervention in tumor immune evasion.

**Supplementary Information:**

The online version contains supplementary material available at 10.1007/s00018-024-05285-1.

## Introduction

Natural killer (NK) cell-based cancer therapy has gained significant attention as a promising therapeutic platform in the field of immunotherapy [1]. NK cells exert their cytotoxic functions through recognizing target cells with the NK group 2-member D (NKG2D) receptor. The interaction between the NKG2D receptor and its ligands (NKG2DL) has attracted considerable attention in the field of cancer immunotherapy. This is mainly attributed to the tumor cells' selective expression of "stress-induced ligands" and the remarkable ability of the NKG2D receptor to activate NK cells [2]. However, accumulating evidence demonstrates that desensitization and functional impairment of NK cells induced by persistent exposure to NKG2D ligands is one of the essential mechanisms involved in tumor immune evasion [3]. Multiple clinical studies have demonstrated that measuring serum levels of NKG2D ligands can independently predict the prognosis of patients with hematologic and solid malignancies [4–6]. Soluble NKG2D ligands derived from tumor cells have been widely suggested to be linked with poor clinical prognosis in cancer patients, largely limiting the therapeutic efficacy of NK cells [7, 8]. Understanding the underlying mechanism of NKG2D-NKG2DL-induced NK cell desensitization may help address this clinical challenge effectively. However, the factors regulating this desensitization process remain largely unknown.

The NKG2D receptor has the ability to recognize a wide array of ligands with varied structures. In humans, it specifically recognizes the MHC I Chain-related molecules A and B (MICA and MICB) and the six cytomegalovirus UL16-binding proteins (ULBP1-6) [7]. MICA, a major ligand of NKG2D, is a widely utilized target in NK anti-tumor therapy [9, 10]. Enhanced expression of MICA on a variety of tumor cells has been observed, facilitating the activation of NK cell effector functions via the MICA-NKG2D axis, consequently bolstering anti-tumor efficacy [11, 12]. However, the shedding of MICA and the prolonged exposure to the soluble MICA (sMICA) environment lead to desensitization and dysfunctional of NK cells, becoming a crucial therapeutic target in tumor immune evasion [13, 14].

In this study, we established a model of sMICA-induced NK cell desensitization and performed RNA sequencing to elucidate regulatory factors involved in tumor immune evasion. Notably, we identified circular RNA *circARAP2* (hsa_circ_0069396) as a key regulatory molecule involved in this biological process. Circular RNAs (circRNAs) play a crucial role in tumor metastasis through diverse mechanisms [15, 16], but their role in NK cell desensitization remains unknown. Circular RNA hsa_circ_0069399, derived from the same parental gene, has been shown to enhance esophageal squamous cell carcinoma progression by modulating the microRNA-761/ Forkhead box M1 (FOXM1) axis. This axis regulates the stemness and endothelial-mesenchymal transition [17]. Thus, in this study we launched a series of functional and mechanistic assays to elucidate the role of *circARAP2* in sMICA-induced NK cell desensitization. We show that loss of *circARAP2* alleviates NKG2D endocytosis and NK cell desensitization. Mechanistically, *circARAP2* exerts its function in the cell nucleus by regulating target genes through protein interactions. This study firstly reports the regulatory role of *circARAP2* in NK cell desensitization, suggesting a novel therapeutic target for improving NK cell function in tumor immune evasion.

## Results

### CircARAP2 is a key factor in sMICA-induced NK cell desensitization

**Fig. 1:**
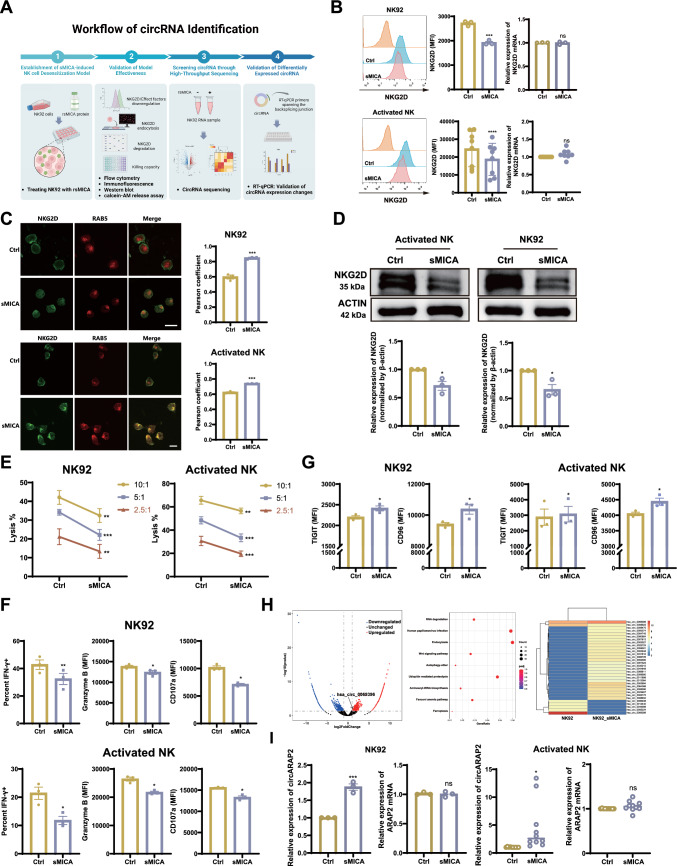
*CircARAP2* is upregulated in sMICA-induced NK cell desensitization. **A** The workflow of profiling circRNAs associated with sMICA-induced NK cell desensitization. **B** Flow cytometry and RT-qPCR analysis of NKG2D expression on NK92 and human primary activated NK cells treated with rsMICA for 24 h. n = 3 for NK92 cells, n = 8 for activated NK cells; Unpaired two-tailed t-test for RT-qPCR analysis, paired two-tailed t-test for flow cytometry analysis. **C** Left panel, Representative IF image of NKG2D and RAB5 on NK92 (Scale bar, 20 μm) and human primary activated NK (Scale bar, 10 μm) cells treated with rsMICA; Right panel, Quantification of Pearson coefficient of colocalization in pairs NKG2D/RAB5 on NK92 and human primary activated NK cells following rsMICA treatment (Repeats = 3, unpaired two-tailed t-test). **D** Immunoblot of NKG2D in both NK92 and human primary activated NK cells treated with rsMICA. Data represent three independent experiments, unpaired two-tailed t-test. **E** The calcein-AM release assay was used to assess the cytotoxicity of NK92 and human primary activated NK cells following rsMICA treatment. Data are presented of three independent experiments, two-way ANOVA. **F** Flow cytometry was used to assess the IFN-γ, Granzyme B and CD107a expression in both NK92 and human primary activated NK cells treated with rsMICA. Data are presented of three independent experiments, paired two-tailed t-test. **G** Flow cytometry was used to assess the expression of inhibitory receptors TIGIT and CD96 in NK92 and human primary activated NK cells with rsMICA treatment. Data are presented of three independent experiments, paired two-tailed t-test. **H** Volcano plot showing differentially expressed circRNAs in NK92 samples with or without rsMICA treatment (Left panel). KEGG analysis showing enrichment of differentially expressed circRNAs in the endocytosis pathway (Middle panel). Heatmap showing changes in expression levels of circRNAs enriched in the endocytosis pathway (Right panel). **I** Expression of *circARAP2* and linear *ARAP2* in both NK92 (n = 3) and primary activated NK cells (n = 10) after treatment with rsMICA. Unpaired two-tailed t-test was used for the comparison of two groups. (*ns* no significance, *P < 0.05, **P < 0.01, ***P < 0.001, ****P < 0.0001)

The MICA/B-NKG2D signal axis plays a pivotal role in tumor immune surveillance, with MICA being a critical ligand for NKG2D and widely expressed in various tumor cells [9–12]. To identify factors that are involved in tumor immune escape, we established a sMICA-NKG2D-induced NK cell desensitization model (Fig. 1A). In this model, NK92 cells were treated with varying concentrations of recombinant soluble MICA protein (rsMICA). This rsMICA protein was the extracellular domain of human recombinant MICA protein, which contains amino acids Glu 24-Gln 308, sourced from Acro Biosystems (MIA-H5221). Flow cytometry was employed to assess NKG2D levels on the cell surface. As the concentration of rsMICA increased, we noted a clear trend of increased NKG2D downregulation on NK92 cell surface (Fig. S1A). We determined the optimal concentration of rsMICA (2000 pg/ml) for subsequent experiments and treated both NK92 cell line and human primary activated NK cells with rsMICA. Flow cytometry revealed consistent downregulation of cell surface NKG2D, while the RNA levels of NKG2D remained unchanged (Fig. 1B). We performed immunofluorescence to track NKG2D and found that NKG2D was primarily localized on the cell membrane in the control group. However, following rsMICA treatment, we observed a noticeable internalization of NKG2D in both NK92 and human primary activated NK cells, which was co-localized with RAB5, an intracellular marker of early endocytosis (Fig. 1C). These data suggest that consistent treatment of sMICA induces NKG2D endocytosis in NK cells.

In addition, we observed varying degrees of increases in the expression levels of endosomal proteins, including early endosome antigen 1 (EEA1), Ras-Related Protein (RAB) 5, RAB7, and lysosomal-associated membrane protein 1 (LAMP1) in rsMICA-treated NK92 cells (Fig. S1B). The results indicated the involvement of both the early and late endosomal pathways, as well as the lysosomal pathway. We then utilized an endocytosis-specific inhibitor dynasore to validate the role of rsMICA-induced NKG2D internalization. Following the pretreatment of endocytosis inhibitor dynasore, we observed a substantial blockage of rsMICA-induced NKG2D endocytosis in NK92 cells (Fig. S1C). Meanwhile, we also obtained consistent results in human activated NK cells (Fig. S1D). Furthermore, we observed that the internalized NKG2D underwent degradation in both NK92 and human activated NK cells (Fig. 1D). Utilizing lysosomal inhibitor NH_4_CL and proteasomal inhibitor MG132, we demonstrated that the internalized NKG2D was degraded through the lysosomal pathway in both NK92 (Fig. S1E) and human activated NK cells (Fig. S1F). In addition, we conducted a thorough exploration of the rsMICA-induced NK cell desensitization phenotype and observed that rsMICA treatment significantly attenuated the NK cell's cytotoxic function (Fig. 1E) and the expression of key effector molecules such as IFN- γ, Granzyme B, and CD107a (Fig. 1F). Previous studies have revealed that persistent NKG2D stimulation can also cross-influence other unrelated receptors on NK cell surface, further promoting NK cell desensitization and dysfunction [18, 19]. In our study, we observed an increase in the expression of the inhibitory receptors TIGIT and CD96 on the surface of both NK92 and human primary activated NK cells following rsMICA treatment, further indicating the desensitization effect of NK cells (Fig. 1G).

Our previous studies have shown in-depth investigation into tumor-derived circRNAs [15, 16], and multiple studies have reported the regulatory function of tumor-derived circRNAs in tumor immune escape [20–22]. However, there is limited understanding of the biological functions of immune cell-derived circRNAs. Therefore, we subsequently focused on exploring the regulatory role of circRNAs in sMICA-induced NK cell desensitization. To identify factors that regulate the endocytosis of NKG2D in this model, we performed RNA sequencing on rsMICA-treated NK92 cell samples (Fig. 1A). Differentially-expressed circRNAs were screened at log2 |Foldchange|> 1 and p-value < 0.05. According to KEGG analysis, there was a significant enrichment of differentially expressed circRNAs in the endocytosis pathway (Fig. 1H). We conducted cluster analysis on circRNAs enriched in the endocytosis pathway and validated their expression levels by RT-qPCR. Notably, our findings indicate that *circARAP2* (has_circ_0069396) was upregulated in both NK92 and human primary activated NK cells following rsMICA treatment, whereas the mRNA expression of its parental gene, *ARAP2*, remained unchanged (Fig. 1I).

### SRSF1 regulates circARAP2 back-splicing in sMICA-induced NK cell desensitization

**Fig. 2:**
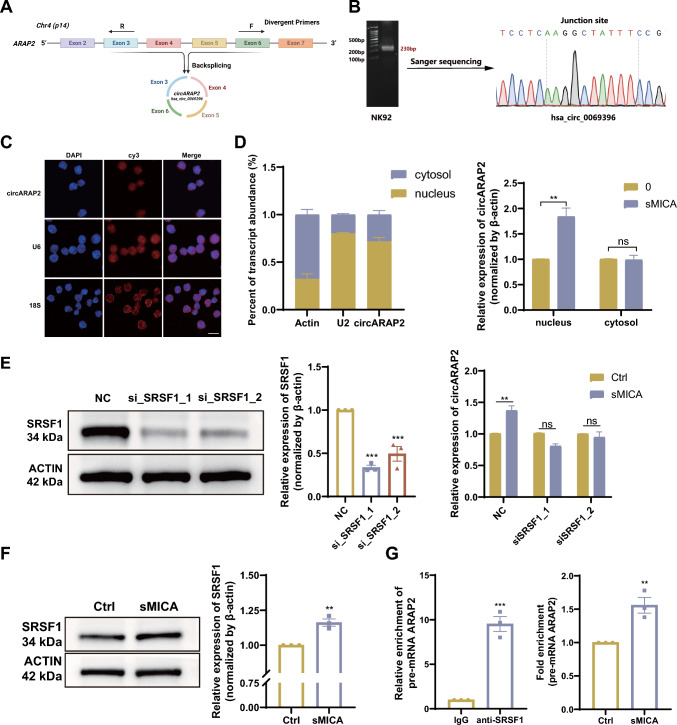
Characteristics of *circARAP2*. **A** Depiction of the annotated genomic region of *ARAP2* and the presumed splicing form of *circARAP2*. Divergent primers were used to amplify the products resulting from back-splicing. **B** The PCR analysis was conducted using the divergent primers Sanger sequencing allowed for the identification of the junction sequence of *circARAP2* in NK92 cells. **C** FISH assays revealed predominant nuclear localization of *circARAP2* (Scale bar, 20 μm). **D** Left panel, The cytoplasmic and nuclear levels of circ*ARAP2* were assessed in NK92 cells, with β-Actin and U2 RNA serving as controls for the cytoplasmic and nuclear compartments, respectively; Right panel, The cytoplasmic and nuclear levels of circ*ARAP2* in both rsMICA-treated and untreated NK92 cells. Data are presented of three independent experiments, two-way ANOVA. **E** The RT-qPCR analysis was conducted to assess *circARAP2* expression in NK92 cells that were transfected with siRNA targeting SRSF1 and control, with or without rsMICA treatment. si_SRSF1_1 and _2 represent two different sets of siRNA. Data are presented of three independent experiments, one/two-way ANOVA. **F** The levels of SRSF1 protein in NK92 cells were measured with or without rsMICA treatment. Data are presented of three independent experiments, unpaired two-tailed t-test. **G** Left panel, RNA immunoprecipitation assay identifying binding between *pre-mRNA ARAP2* and SRSF1; Right panel, The comparison of binding between *pre-mRNA ARAP2* and SRSF1 in NK92 cells with or without rsMICA treatment. Data are presented of three independent experiments, unpaired two-tailed t-test. (*ns* no significance, **P < 0.01, ***P < 0.001)

*CircARAP2* is a circular RNA comprised of exons 3 to 6 of the *ARAP2* gene, spanning 582 bp in length (Fig. 2A). Sanger sequencing was performed to determine the back-spliced junction region of *circARAP2* (Fig. 2B). To confirm whether *circARAP2* was back-spliced, we designed divergent and convergent primers for amplifying the transcription of *circARAP2* and linear *ARAP2* mRNA, respectively. Our findings revealed that *circARAP2*, unlike linear *ARAP2*, displayed specific amplification only in cDNA samples using divergent primers. Conversely, showed amplification in both gDNA and cDNA samples with convergent primers. The result indicates that *circARAP2* is a back-spliced circRNA (Fig. S2A). Furthermore, RNA reverse transcription was conducted using oligo dT primers and random hexamers. Our findings indicated successful detection of linear *ARAP2* transcripts using both primer types. However, *circARAP2* was barely detectable with oligo dT primers, which is in line with the characteristics of circRNAs that lack poly-A tails (Fig. S2B). RNase R assay revealed that *circARAP2* was more tolerated than its linear *ARAP2* counterpart, confirming its circular structure (Fig. S2C). To assess the stability of *circARAP2*, an actinomycin D test was carried out. The findings revealed that *circARAP2* exhibited a longer metabolic half-life compared to linear *ARAP2* (Fig. S2D). These results indicated that *circARAP2* displayed characteristics consistent with back-spliced circRNA.

To investigate the subcellular distribution of *circARAP2*, we performed fluorescence in situ hybridization (FISH) and cytoplasm-nucleus separation assays. Using a Cy3 probe specific to *circARAP2*, we confirmed that *circARAP2* was predominantly localized in the nucleus (Fig. 2C). RT-qPCR analysis of the cytoplasm-nucleus separation assay carried out in NK92 cells further confirmed the nuclear localization of *circARAP2* (Fig. 2D Left). Furthermore, following the separation of cytoplasmic and nuclear fractions in both rsMICA-treated and untreated NK92 cells, we observed a significant upregulation of *circARAP2* in the nucleus of rsMICA-treated NK92 cells, whereas the expression level of *circARAP2* in the cytoplasm remained relatively unchanged (Fig. 2D Right). Meanwhile, we also observed consistent upregulation of *circARAP2* in the nucleus of human primary activated NK cells treated with rsMICA (Fig. S2E). According to the data obtained, there is a predominant nuclear location of *circARAP2*.

To explore the potential mechanisms underlying the increased expression of *circARAP2*, we concentrated on splicing factors that could potentially participate in the back-splicing process. Based on information from circInteractome [23], potential associations were identified between two RNA splicing factors, Eukaryotic initiation factor 4A-III (eIF4A3) and Serine/Arginine splicing factor 1 (SRSF1), and the upstream or downstream regions of *pre-ARAP2*. *Pre-ARAP2* represents the immature state of *circARAP2* prior to the back-splicing process (Fig. S2F). To validate their regulatory roles, siRNAs were used to target these two factors and were transfected into NK92 cells. We found that SRSF1 had the strongest influence on *circARAP2* expression, whereas the expression of mRNA-*ARAP2* remained unaffected (Fig. S2G). The protein level of SRSF1 was moderately increased in rsMICA-treated NK92 cells, and loss of SRSF1 suppressed the increased expression of *circARAP2* induced by rsMICA (Fig. 2E and F). We then confirmed the interaction between SRSF1 and *pre-ARAP2* at the predicted binding site through the RNA immunoprecipitation assay (RIP) (Fig. 2G Left). Subsequently, we conducted the RIP assay on NK92 cells following rsMICA treatment and observed an increase in the binding of SRSF1 to *pre-ARAP2*. This suggests the involvement of SRSF1 in the back-splicing and generation of *circARAP2* in sMICA-induced NK cell desensitization process (Fig. 2G Right).

### Disrupting circARAP2 alleviates sMICA-induced NK desensitization

**Fig. 3:**
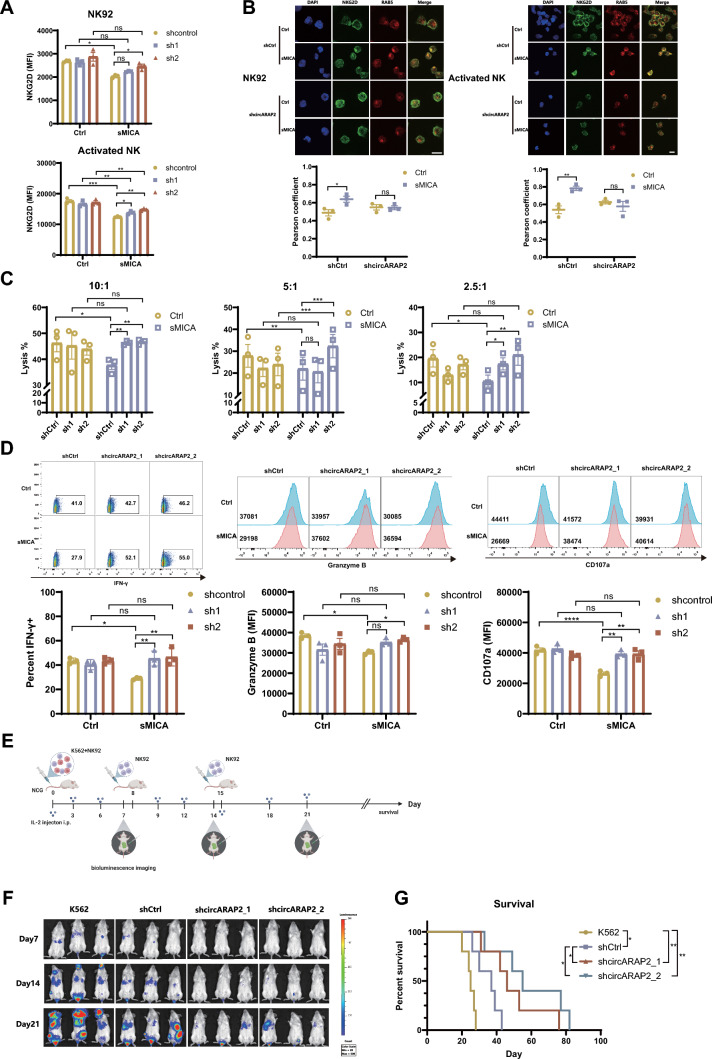
Downregulation of *circARAP2* alleviates sMICA-induced NK desensitization. **A** Flow cytometric analysis was employed to evaluate the levels of NKG2D expression in NK92 and primary activated NK cells transfected with shCtrl and shcric*ARAP2*, with or without rsMICA treatment. **B** Up panel, Representative IF image of NKG2D and RAB5 on NK92 (Scale bar, 20 μm) and primary activated NK (Scale bar, 10 μm) cells stably transfected with shCtrl and shcric*ARAP2*, with or without rsMICA treatment; Down panel, The corresponding quantification of colocalization in pairs NKG2D/RAB5 on NK92 and activated NK cells (Repeats = 3, two-way ANOVA). **C** The calcein-AM release assay was utilized to assess the cytotoxicity of NK92 cells, which were transfected with either shCtrl or sh*cricARAP2* and treated with or without rsMICA. **D** Flow cytometry was used to assess the IFN-γ, Granzyme B and CD107a expression in NK92 cells transfected with shCtrl or shcric*ARAP2*, with or without rsMICA treatment. **E** Illustration of the tail vein luciferase-labeled K562 model in immunodeficient NCG mice and treatment with NK92 cells. **F** Representative mice from each group were subjected to bioluminescent imaging at Day 7, 14, and 21 to capture tumor images. **G** The survival rates of mice in the four different groups were assessed using a Kaplan–Meier survival curve analysis. For **A**, **C**, **D**, data are presented of three independent experiments, two-way ANOVA. (*ns* no significance, *P < 0.05, **P < 0.01, ***P < 0.001, ****P < 0.0001)

To determine the function of *circARAP2* in sMICA-induced NK cell desensitization, we disrupted *circARAP2* expression in both NK92 and human primary activated NK cells by small hairpin RNAs (shRNAs) that specifically target the circular junction of *circARAP2*. Notably, expression of sh*circARAP2s* only disrupted the expression of *circARAP2*, but not its linear counterpart, as demonstrated by RT-qPCR (Fig. S3A). After expressing sh*circARAP2s* in NK92 and human primary activated NK cells, we found that rsMICA-induced NKG2D downregulation was alleviated in *circARAP2*-knockdown NK cells, as compared with NK cells expressing paired control shRNAs (Fig. 3A). Consistently, the results from immunofluorescence further revealed that *circARAP2*-knockdown effectively inhibited rsMICA-induced NKG2D internalization in NK92 and human primary activated NK cells (Fig. 3B). Western blot also indicated the inhibition effect of *circARAP2*-knockdown on rsMICA-induced NKG2D degradation in NK92 cells (Fig. S3B). In addition, our results showed that rsMICA treatment impaired NK function by reducing their degranulation and killing capacity. However, we observed a significant improvement in NK cell function upon silencing *circARAP2*, manifested by rescued NK92 cell killing of K562 tumor cells (Fig. 3C) and expression of effector molecules such as IFN-γ, Granzyme B, and CD107a in both rsMICA-treated NK92 (Fig. 3D) and human activated NK cells (Fig. S3C). In addition to the in vitro experiments, we further investigated the efficacy of *circARAP2* knockdown in alleviating sMICA-induced NK cell dysfunction in vivo. K562 are lymphoblast cells isolated from a patient with chronic myelogenous leukemia (CML), known for their high expression of MICA and sensitivity to NK cell cytotoxicity [24]. Moreover, it has been reported that Chronic myeloid leukemia (CML) patient serum displays elevated levels of sMICA [4]. In our study, we collected the supernatant of K562 cell cultures at various time intervals for ELISA detection and observed that the production of sMICA by K562 cells gradually increased over time (Fig. S3D Left). Furthermore, we employed the supernatant of K562 cells collected at different time points to treat NK92 cells for 24 h. We found that, as the concentration of sMICA increased, the downregulation of NKG2D on NK cells became increasingly significant (Fig. S3D Right). Therefore, we used K562 cells capable of producing sMICA to simulate the sMICA-induced NK cell desensitization model in NCG mice, and preliminarily determined the effect on the anti-tumor activity of NK cells through tumor development. In our study, we injected NK92 cells that had undergone transfection with sh*circARAP2s* and luciferase-expressing K562 cells into NCG mice through the tail vein (Fig. 3E). Bioluminescence imaging in mice demonstrated that knocking down *circARAP2* effectively suppressed tumor progression (Fig. 3F), and the survival curve of tumor-bearing mice indicated that *circARAP2* knockdown significantly prolongs their survival time (Fig. 3G). These results indicate that NK92 cell desensitization induced by sMICA may be the main reason for tumor progression and *circARAP2* is a promising therapeutic target that warrants further investigation.

### RAB5A is the downstream target gene of circARAP2 in sMICA-induced NK desensitization

**Fig. 4:**
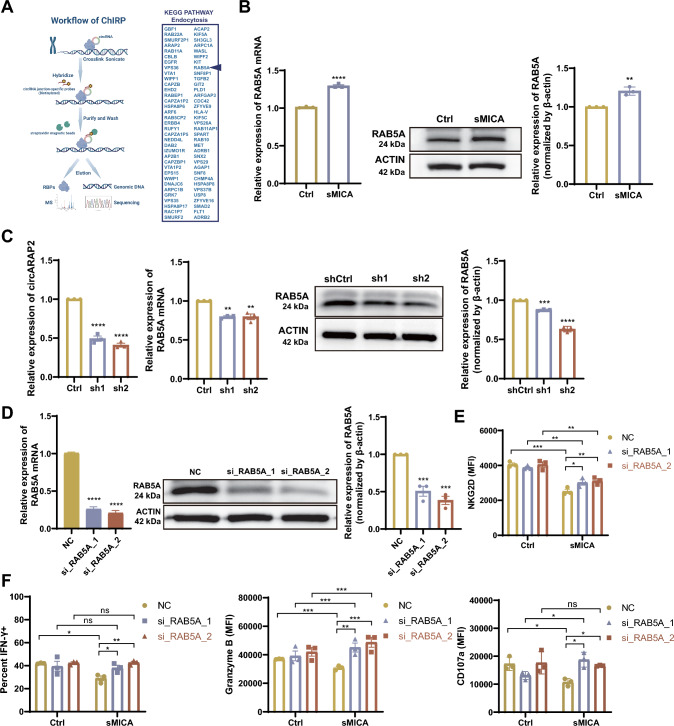
*RAB5A* is the target gene of *circARAP2* in sMICA-induced NK cell desensitization. **A** Left panel, Schematic illustrating the methodology used to identify the binding target genes of *circARAP2*. Right panel, ChIRP-sequencing identified binding target genes enriched in the endocytosis pathway. **B** Immunoblot and RT-qPCR showing the levels of *RAB5A* expression in NK92 cells with or without rsMICA treatment. Data are presented of three independent experiments, unpaired two-tailed t-test. **C** Immunoblot and RT-qPCR were employed to evaluate the expression of *RAB5A* with or without *circARAP2* knockdown. **D** We assessed the efficiency of *circARAP2* knockdown using Immunoblot and RT-qPCR. **E** Flow cytometry was employed to assess NKG2D expression in NK92 cells that were transfected with siRNA targeting *RAB5A* and control, with or without rsMICA treatment. **F** Flow cytometry was used to assess the IFN-γ, Granzyme B and CD107a expression in NK92 cells transfected with siRNA targeting *RAB5A* and control, with or without rsMICA treatment. For **C** and **D**, data are presented of three independent experiments, one-way ANOVA. For **E** and **F**, data are presented of three independent experiments, two-way ANOVA. (*ns*: no significance, *P < 0.05, **P < 0.01, ***P < 0.001, ****P < 0.0001)

To delineate the mechanisms underlying the role of *circARAP2* in NK cell desensitization, we conducted the Chromatin Isolation by RNA Purification (ChIRP) assay followed by DNA sequencing (DNA-seq). We designed two biotin-labeled probes that specifically target the back-splicing junction site of *circARAP2*. These probes could capture *circARAP2* and its associated DNA fragments by binding to streptavidin. The captured genome DNAs were used for high-throughput sequencing to obtain downstream target genes for *circARAP2*, with a focus on genes in the endocytosis pathway (Fig. 4A). Among them, *RAB5A* was not only interacted with *circARAP2,* but was also upregulated at both the RNA and protein levels in rsMICA-treated NK92 cells (Fig. 4B, part of Fig. S1B). We thus focused on the role of *RAB5A* as a potential downstream target gene of *circARAP2*. *RAB5A* (the most important subtype of RAB5) is a small molecule RAB GTPase that mainly localizes in early endosomes, with functions and mechanisms that are well-understood. It is essential in regulating the fusion between phagosomes and early endosomes, making it a key limiting component in the initial stages of endocytosis [25]. The results of the immunofluorescence experiment revealed a substantial co-localization between endocytic NKG2D and RAB5A in rsMICA-treated NK92 and human activated NK cells (Fig. 1C). To confirm the regulatory function of *circARAP2* on *RAB5A* transcription, we performed shRNA knockdown of *circARAP2* in NK92 cells, which led to decreased RNA and protein levels of *RAB5A* (Fig. 4C). To confirm the role of *RAB5A* in sMICA-induced NK cell desensitization, two siRNA sequences were designed to induce the knockdown of *RAB5A* (Fig. 4D). We found that loss of *RAB5A* inhibited rsMICA-induced NKG2D downregulation (Fig. 4E) and internalization (Fig. S3E), thereby rescuing the expression of IFN-γ, Granzyme B, and CD107a in NK92 cells (Figs. 4F and S3F).

### CircARAP2 interacts with CTCF to regulate RAB5A expression in NK cells

We then conducted RNA pull-down experiments to investigate if *circARAP2* functions through the interaction with chromatin factors. Specifically, we used an in vitro transcribed biotin-*circARAP2* probe to capture the proteins and analyzed them using silver staining and mass spectrometry (Fig. S4A). Based on the protein information obtained through mass spectrometry and CHIP-sequencing data from UCSC Genome Browser, we speculated that the binding of *circARAP2* to CCCTC-binding factor (CTCF) could potentially regulate the transcription of the target gene *RAB5A* (Figs. S4B and S5A). As a versatile nuclear factor, CTCF performs multiple roles in various biological processes, including transcriptional activation or repression, binding as an insulator factor, and regulation of genomic imprinting [26].

**Fig. 5:**
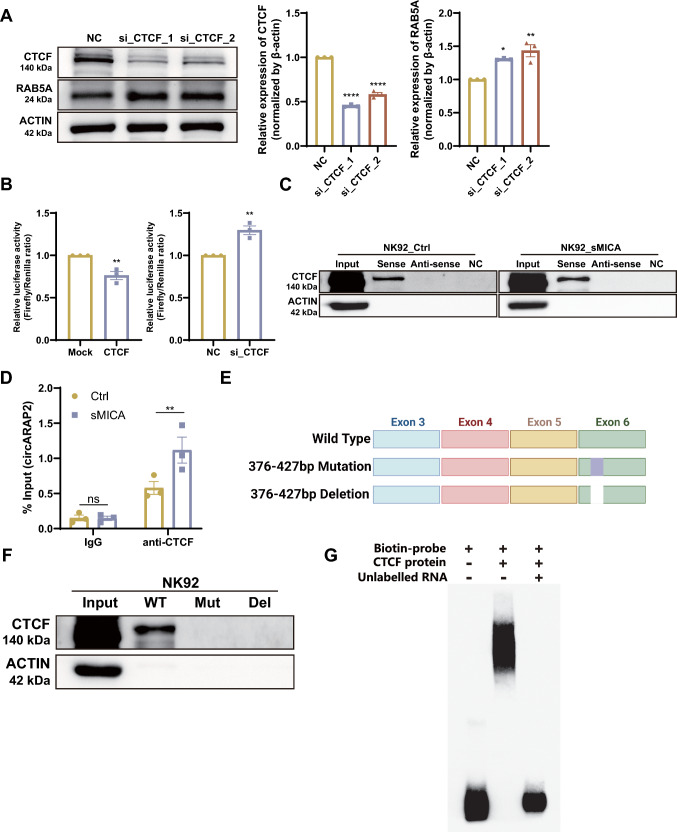
*CircARAP2* interacts with CTCF to regulate *RAB5A* expression in NK cells. **A** Immunoblot was used to determine the expression of *RAB5A* with or without CTCF knockdown. si_CTCF_1 and _2 represent two different sets of siRNA. Data are presented of three independent experiments, one-way ANOVA. **B** The Dual-Luciferase Reporter Assay demonstrated alterations in the promoter activity of *RAB5A* in 293 T cells with stable transfection of either CTCF overexpression plasmid or CTCF-specific siRNA. Data are presented of three independent experiments, unpaired two-tailed t-test. **C** Immunoblot analysis of CTCF in protein products from RNA pull-down assays using *circARAP2* specific probes. **D** RT-qPCR analysis of *circARAP2* in RNA samples after RIP assay by anti-CTCF antibody. Data are presented of three independent experiments, two-way ANOVA. **E** Schematic illustrating the wild-type, mutant and deleted *circARAP2* probes utilized in the RNA pull down assay. **F** Immunoblot analysis of CTCF in protein products from RNA pull-down assays using wild-type, mutant and deleted *circARAP2* probes. **G** RNA EMSA was conducted to investigate the interaction between CTCF and the 376-427 bp of *circARAP2* in NK92 cells. (*ns* no significance, *P < 0.05, **P < 0.01, ****P < 0.0001)

To confirm the regulatory function of CTCF on *RAB5A* transcription, we performed siRNA knockdown of CTCF in NK92 cells, which resulted in a modest elevation in the protein level of *RAB5A* (Fig. 5A). The impact of CTCF on the transcriptional activity of the *RAB5A* promoter was further corroborated by the Dual-Luciferase Reporter Assay. We generated a fluorescein reporter plasmid containing *RAB5A* promoter region and conducted the Dual-Luciferase Reporter Assay in 293 T cells. It was observed that knockdown of CTCF significantly increased *RAB5A* gene promoter activity, while overexpression of CTCF led to a decrease in *RAB5A* promoter activity (Fig. 5B). These results indicated that *circARAP2* and CTCF have opposite functions in the control of *RAB5A* transcription.

We then utilized the catRAPID platform to predict the interaction between *circARAP2* and CTCF proteins. After entering the *circARAP2*-582 bp sequence and CTCF 727 amino acid sequence into the catRAPID graphic program, we obtained an interaction propensity of 8, with a discriminative power of 28%. Further catRAPID fragment analysis indicated the highest probability of binding near the sixth exon sequence (especially 376–427 bases) (Fig. S4C). To confirm the interaction between *circARAP2* and CTCF, we conducted Western blot analysis on the proteins extracted from the RNA pull-down assay. The data obtained indicated that CTCF could bind with *circARAP2* in both rsMICA-treated and untreated NK92 cells (Fig. 5C). The RNA RIP assay demonstrated a higher degree of binding between CTCF and *circARAP2* in rsMICA-treated NK92 cells compared to untreated cells (Fig. 5D). Furthermore, we utilized a combination of immunofluorescence and RNA FISH to confirm that CTCF and *circARAP2* were co-localized significantly with DAPI in the nucleus of NK92 cells (Fig. S4D).

To further elucidate the binding region between *circARAP2* and CTCF, we performed an RNA pull-down assay using NK92 extracted proteins with wild-type, mutant (376-427 bp) and deleted (376-427 bp) *circARAP2* probes (Fig. 5E). The results indicated that *circARAP2* bound to CTCF via its 376–427 bases (Fig. 5F). The data of the RNA EMSA assay further supported that *circARAP2* specifically interacted with CTCF at its 376–427 bases (Fig. 5G).

### CircARAP2 suppresses the recruitment of CTCF-PRC2 and erases histone suppression in the RAB5A promoter

**Fig. 6:**
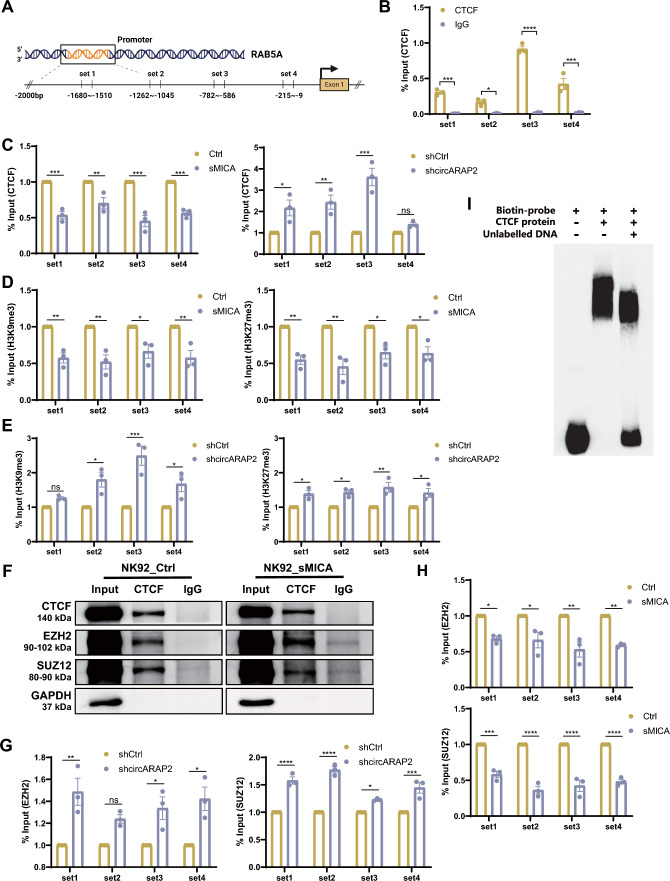
*CircARAP2* suppresses CTCF recruitment to the *RAB5A* promoter. **A** Schematic illustrating the set1-4 primers used in CHIP to detect the protein binding to *RAB5A* promoter region. **B** ChIP and RT-qPCR assays demonstrated the presence of endogenous CTCF binding on *RAB5A* promoter region. **C** ChIP and RT-qPCR assays were conducted to evaluate the alterations in CTCF binding to the promoter region of *RAB5A* in NK92 cells under different conditions, including rsMICA treatment and stable transfection of shCtrl or shcric*ARAP2*. **D** ChIP and RT-qPCR assays were conducted to assess the variations in the binding of H3K9me3 and H3K27me3 to the promoter region of *RAB5A* in NK92 cells with or without rsMICA treatment. **E** The same indicators used in **D** were detected in NK92 cells with stable transfection of shCtrl and shcric*ARAP2*. **F** Co-IP assays revealed an interaction between CTCF and PRC2 complex components EZH2 and SUZ12. **G** ChIP and RT-qPCR assays indicating the alterations in the binding of EZH2 and SUZ12 to the promoter region of *RAB5A* in NK92 cells stably transfected with shCtrl and shcric*ARAP2*. **H** ChIP and RT-qPCR assays were carried out to evaluate the alterations in the binding of EZH2 and SUZ12 to *RAB5A* promoter in NK92 cells with or without rsMICA treatment. **I** The EMSA assay indicating the competition between DNA (196 bp) and RNA (52 bp) probes for binding to CTCF. For **B**–**H**, data are presented of three independent experiments, two-way ANOVA. (ns: no significance, *P < 0.05, **P < 0.01, ***P < 0.001, ****P < 0.0001)

We further examined the mechanism by which *circARAP2* coordinates CTCF to regulate the *RAB5A* promoter. First, the ChIP-sequencing data acquired from UCSC Genome Browser revealed a significant enrichment of CTCF within the promoter region of *RAB5A* in human (Fig. S5A). Second, we conducted a ChIP assay using four different primer sets (Sets 1–4) to examine the CTCF binding site in the *RAB5A* promoter region (Fig. 6A). The ChIP and real-time quantitative PCR results revealed that CTCF bound mainly to the Set 3 primer site in the *RAB5A* promoter (Fig. 6B). Third, we conducted CHIP-qPCR assays to evaluate the impact of *circARAP2* on CTCF binding to the *RAB5A* promoter in NK92 cells. The results showed that the rsMICA-induced upregulation of *circARAP2* led to a reduction in CTCF binding to the *RAB5A* promoter, in parallel with the alleviation of CTCF-mediated repression and subsequent transcriptional activation of *RAB5A* (Fig. 6C Left). In contrast, knockdown of *circARAP2* caused an increase in the enrichment of CTCF within the *RAB5A* promoter region, resulting in an amplification of the inhibitory of CTCF on *RAB5A* transcription (Fig. 6C Right).

Our previous studies have shown that CTCF functions by tethering the suppressor of zeste 12 protein homolog (SUZ12), a docking factor of the polycomb repressive complex-2 (PRC2), to the target promoter. The PRC2 factor enhancer of zeste homolog 2 (EZH2) induces histone H3 lysine 27 methylation to suppress the imprinted allele [27–29]. We thus tested if *circARAP2* used a similar mechanism to regulate the *RAB5A* function in NK cells. Using the UCSC Genome Browser, a clear enrichment of repressive chromatin markers H3K9me3 and H3K27me3 was identified surrounding CTCF binding sites in *RAB5A* promoter (Fig. S5B). To confirm whether CTCF impacts chromatin accessibility by influencing histone methylation modifications and therefore regulating *RAB5A* transcription, we performed a CHIP assay following CTCF knockdown in NK92 cells. Our results revealed a significant reduction of H3K27me3 and H3K9me3 enrichment in the promoter region of *RAB5A*, suggesting that CTCF promotes H3K27me3 and H3K9me3 enrichment and thereby suppresses *RAB5A* transcription (Fig. S5C). To clarify the function of *circARAP2* in this process, we conducted another CHIP-qPCR analysis on rsMICA-treated and untreated NK92 cells, as well as on NK92 cells with and without *circARAP2* knockdown. The results revealed a decrease of H3K9me3 and H3K27me3 enrichment in the promoter region of *RAB5A* following rsMICA treatment (Fig. 6D), whereas knockdown of *circARAP2* led to increased H3K9me3 and H3K27me3 enrichment in the same region (Fig. 6E). We have validated the interaction between CTCF and EZH2, as well as SUZ12, through co-immunoprecipitation assays in both untreated and rsMICA-treated NK92 cells (Fig. 6F). We then conducted a CHIP assay to validate the observed enrichment of EZH2 and SUZ12 near CTCF binding sites in the promoter region of *RAB5A*, as indicated by UCSC Genome Browser (Fig. S5D). The results showed an increase in EZH2 and SUZ12 enrichment in the promoter region of *RAB5A* after *circARAP2* knockdown (Fig. 6G), while rsMICA treatment resulted in decreased EZH2 and SUZ12 enrichment in the same region (Fig. 6H). These data indicated that the *circARAP2*-CTCF-PRC2-*RAB5A* axis acts as a primary regulator in sMICA-induced NK cell desensitization.

**Fig. 7:**
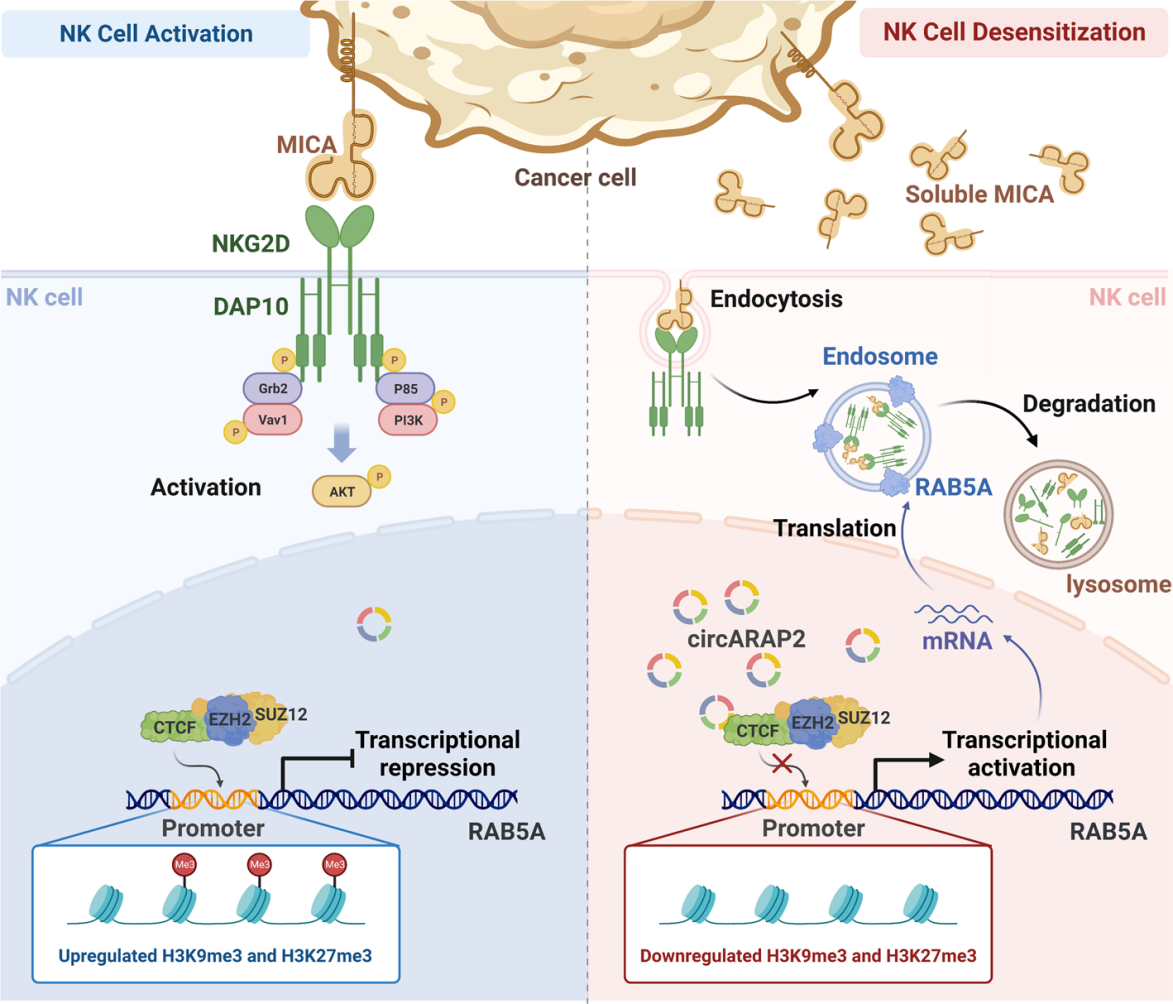
A proposed model for *circARAP2*-mediated regulation in sMICA-induced NK cell desensitization. The NKG2D-NKG2DL axis is typically responsible for activating NK cells and making cancer cells vulnerable to lysis mediated by NK cells. However, extended exposure to sMICA causes endocytosis and subsequent degradation of NKG2D receptors on NK cell surface, which results in desensitization and low responsiveness towards cancer cells. CTCF-PRC2 complex is able to suppress the transcription of endocytic-related protein RAB5A through regulation of chromatin remodeling. The upregulated *circARAP2* in sMICA-exposed NK cells can suppress CTCF function by suppressing the enrichment of CTCF to *RAB5A* promoter, thus regulating NKG2D endocytosis

To further elucidate the underlying regulatory mechanism of *circARAP2* in modulating CTCF enrichment, we included the *RAB5A*-set3 DNA probe (196 bp, −782 to  −586 bases) in the EMSA assay. Our observations revealed that it competes with *circARAP2* (52 bp, 376–427 bases) for binding to CTCF (Fig. 6I). The results of the in vitro triplex formation assay indicated that there was no interaction between Cy3-labeled *RAB5A* probe (196 bp) and Cy5-labeled *circARAP2* probe (52 bp). This finding suggests that *circARAP2* does not hinder CTCF enrichment by binding to the corresponding DNA promoter region (Fig. S5E). Therefore, we speculate that *circARAP2* may regulate the interaction between the CTCF-PRC2 complex and the promoter region of *RAB5A* through two distinct mechanisms. This includes altering the spatial conformation of CTCF or competing with promoter DNA sequence for the binding domain on CTCF. Consequently, this leads to hindered enrichment of CTCF and ultimately affects the regulation of *RAB5A* (Fig. 7).

## Discussion

The NKG2D-NKG2DL axis-induced desensitization of NK cells is widely recognized as a major contributor to tumor immune evasion and a crucial factor that limits the clinical efficacy of NK cell therapy [30]. NK cell desensitization can occur when the activating receptors represented by NKG2D are persistently stimulated by ligands on tumor cell surfaces without simultaneous engagement of inhibitory receptors, inducing a hyporesponsive state [31, 32]. Previously published papers have provided strong evidence that both soluble and membrane-bound NKG2D ligands, including MICA, can promote receptor down-modulation [33–35]. From a clinical perspective, numerous studies have shown a positive correlation between soluble MICA levels in the serum of cancer patients and their poor prognosis, indicating that it is an independent prognosis predictor and promising therapeutic target in cancer patients [14, 36–38].The shed NKG2D ligands, such as the soluble MICA and MICB, can engage NKG2D on NK cells, resulting in systemic NK cell dysfunction [39, 40]. Additionally, recent studies have reported that the desensitization of NK cells induced by NKG2D ligands may be primarily mediated through receptor-mediated endocytosis [41, 42]. Specifically, engagement of NKG2D by MICA in human NK cells induces Vav1 phosphorylation and activates PI3K, initiating intracellular signaling pathways that drive effector functions. However, sustained MICA engagement leads to c-Cbl ubiquitin ligase activation and ubiquitination of DAP10, which leads to internalization of NKG2D and subsequent lysosomal degradation, ultimately resulting in hyporesponsiveness to target cells [43–46]. The specific pathways and mechanisms of membrane-bound MICA and soluble MICA inducing NK cell desensitization might be different. In this study, we established a soluble MICA-induced NK cell desensitization model to provide insights into the challenges posed by soluble MICA in the serum of clinical tumor patients and its adverse prognostic implications. We confirmed that persistent exposure to excessive soluble MICA leads to the internalization of NKG2D and subsequent lysosomal degradation, impairing the function of NK cells and promoting tumor immune escape. In the future, we will further conduct explorations on the specific similarities and differences between membrane-bound MICA and soluble MICA induced NK cell desensitization.

In the subsequent studies, we focused primarily on the crucial RNA molecules involved in the modulation of sMICA-induced NK cell desensitization, and we identified that numerous circRNA expressions were significantly altered during this process. As investigations into circRNAs progress, the biological functions of circRNAs have gradually been revealed [47]. The function of circRNAs is closely associated with their cellular location. When present in the cytoplasm, they function as competing endogenous RNAs (ceRNAs) by sponging miRNAs and preventing them from binding to non-coding regions of mRNA, thereby effectively regulating the expression of target genes [48]. When circRNAs are present in the nucleus, they primarily function through interactions with RNA-binding proteins, and they can also directly bind to RNA or DNA to exert corresponding regulatory effects [49–51]. Based on the published reports, our study primarily analyzed the differentially expressed circRNAs associated with the endocytosis pathway in sMICA-treated NK cells. We selected *circARAP2* as our research subject. Although *circARAP2* is not the most significantly differentially expressed circRNA in the volcano plot, our screening results suggest that it could be an important circRNA involved in the regulation of sMICA induced NK cell desensitization. Given that the endocytosis of NKG2D may be the main reason for NK cell dysfunction in the sMICA-induced NK cell desensitization model, we mainly focus on circRNAs related to the endocytosis pathway. We used RT-qPCR to further validate the differentially expressed circRNAs that are enriched in the endocytosis pathway in both NK92 cell line and human primary activated NK cells. Our findings indicate that *circARAP2* is stably up-regulated in NK cells following sMICA treatment. Therefore, we have chosen *circARAP2* as the key subject of our study, reasoning that it might play a regulatory role in the desensitization process of sMICA treated NK cells. Both in vitro and in vivo phenotype experiments indicated that loss of *circARAP2* can alleviate sMICA-induced NK cell desensitization and dysfunction by inhibiting NKG2D endocytosis, thereby enhancing the anti-tumor capacity of NK cells. Incorporating clinical samples into our future studies will provide additional evidence to reveal the role of *circARAP2* in sMICA-induced NK cell desensitization. FISH and cytoplasm-nucleus separation assays both revealed the predominant localization of *circARAP2* within the nucleus of NK cells. Additionally, the expression of *circARAP2* in the nucleus substantially increased after sMICA treatment, indicating its primary function within the nucleus.

The subsequent results of our study indicated that *circARAP2* may play a regulatory role in sMICA-induced NK cell desensitization by regulating CTCF enrichment and chromatin remodeling on the promoter of *RAB5A*. Ras-like proteins in brain (RAB) are small GTPase proteins that exist in the plasma and organelle membranes of eukaryotic cells. They play a crucial role in regulating intracellular trafficking pathways by interacting with specific effector molecules, which includes modulating the formation, transport, docking, adhesion, anchoring, and fusion processes of protein vesicles within cells [52, 53]. *RAB5A* is a subtype of RAB5 (a pivotal member of the RAB family) that mainly localizes within early endosomes. It is commonly utilized as a molecular marker for early endosomes and has a key regulatory function in the initial stage of endocytic vesicle trafficking [25, 54]. RAB5 exists in two different states: GTP-bound (activated) and GDP-bound (inactivated) states. The transition between these two states is a key rate-limiting step in the process of endocytosis [55]. Our findings demonstrated that *RAB5A* knockdown can effectively alleviate sMICA-mediated NKG2D internalization and subsequent NK cell dysfunction. Moreover, we confirmed that *RAB5A* is a downstream target gene of *circARAP2*, as *circARAP2* knockdown led to a decrease in the levels of both RNA and protein expression of *RAB5A*.

CTCF, a nuclear protein containing 11 zinc-fingers, is a well-conserved multifunctional factor that was initially described as a transcriptional regulator interacting with the promoter region of the *c-MYC* gene [56]. Existing reports have revealed that CTCF has multiple functions within cells, such as activating or repressing transcription, inducing X-chromosome inactivation, regulating gene imprinting, and controlling three-dimensional chromatin conformation. As a transcription factor, CTCF can bind to DNA sequences via its diverse zinc finger domains, allowing it to promote or inhibit gene expression [57, 58]. In our present study, we have provided evidence indicating the binding capability of CTCF to *RAB5A* promoter region, resulting in the inhibition of its transcription. Furthermore, by interacting with CTCF, *circARAP2* can hinder CTCF recruitment to the *RAB5A* promoter region, thereby preventing its transcriptional suppression effect. CTCF has been reported to interact with the promoter region of *P53* and prevent the enrichment of chromatin repression markers, such as H3K9me3 and H3K27me3, thereby preserving the transcriptional activity and tumor-suppressing function of P53 in various types of cancer cells [59]. Similarly, CTCF has also been reported to interact with the *MYCN* promoter region and lead to chromatin remodeling, which enhances the enrichment of activating markers like H3K4me2, H3K4me3, and RNA polymerase II while inhibiting the enrichment of repressive markers such as H3K9me3 and H3K27me3 in the promoter region. As a result, the transcriptional activity of the *MYCN* gene is upregulated, leading to increased expression of the *MYCN* gene [60]. In this study, we found that CTCF can promote the enrichment of chromatin suppressive markers H3K9me3 and H3K27me3 in the promoter region of *RAB5A* through interacting with PRC2, thus inhibiting the transcription of *RAB5A*. On the contrary, *circARAP2* was able to promote the transcription of *RAB5A* by inhibiting the enrichment of H3K9me3 and H3K27me3 in *RAB5A* promoter region through the suppression of CTCF-PRC2 complex.

In summary, this study demonstrates that *circARAP2* is a key regulator in sMICA-induced NK cell desensitization. Mechanistically, *circARAP2* disrupts the enrichment and transcriptional repression of CTCF-PRC2 in the promoter region of *RAB5A* by binding to CTCF, thereby promoting NKG2D endocytosis mediated by *RAB5A* and desensitization of NK cells. These findings demonstrate that *circARAP2* holds promise as a prospective biomarker and therapeutic target for sMICA-induced NK cell desensitization, and also highlight the *circARAP2*/CTCF-PRC2/*RAB5A* axis as a novel mechanism for regulating NK cell desensitization and tumor immune evasion.

## Methods

### Cell lines and cell culture

Blood samples from healthy donors were kindly provided by the Jilin Branch of the Red Cross Society of China. Peripheral blood mononuclear cells were collected through density gradient centrifugation and subsequently activated and expanded utilizing the NK Cell Activation/Expansion Kit (130-094-483, Miltenyi Biotec). The cells were cultured in NK MACS Medium (130-114-429, Miltenyi Biotec) added with 5% human AB serum (HP1022HI, Valley Biomed) and 500 IU/mL interleukin (IL)-2 (130-097-743, Miltenyi Biotec). These cells were incubated with PE-anti-human CD56 antibodies (304605, Biolegend, RRID: AB_314447) to determine the purity of primary activated NK cells.

The NK92 cell line was procured from ATCC (American Type Culture Collection) and cultured in α-MEM (1749161, Gibco) added with 12.5% horse serum (Gibco), 12.5% fetal bovine serum (CellMax), 0.1 mM β-mercaptoethanol (M6250, Sigma-Aldrich), 0.2 mM myo-inositol (17508, Sigma-Aldrich), 0.02 mM folic acid (F8758, Sigma-Aldrich), and 100 IU/ml IL-2.

The K562 cell line and HEK-293 T cell line were procured from ATCC and cultured in RPMI 1640 medium (Gibco) and high glucose DMEM medium (Gibco), respectively. Both mediums were added with 10% fetal bovine serum (CellMax).

### Plasmids and transfection

To specifically knockdown *circARAP2*, we designed two small hairpin RNAs (shRNA) that target the back-splicing junction site of *circARAP2*, while leaving the linear mRNA counterpart unaffected. A random shRNA (shCtrl) was employed as the control. These shRNAs were cloned into PLKO.1 vectors, followed by HEK-293 T cell-mediated packaging of lentiviruses.

NK92 and primary activated NK cells were cultured in six-well plates, with 5 × 10^5^ or 2 × 10^6^ cells per well, respectively, using 2 mL of medium supplemented with 8 μg/mL polybrene (40804ES76, Yeasen). Afterwards, the cells were exposed to various lentiviruses for infection and centrifuged at 1800 g for 1 h at room temperature before being placed in culture at 37 °C. The transfection efficiency was evaluated and the cells were utilized at 40 h post-infection.

### RNA interference and transfection

All small interfering RNAs (siRNA) used for knockdown of the target genes (*CTCF*, *RAB5A*, and *SRSF1*), as well as the scrambled siRNA (used as a negative control), were obtained from GenePharma (Shanghai, China). The siRNA sequences can be found in Supplementary Table 1.

The Cell Line Nucleofector Kit R (VCA-1003, Lonza, Basel, Switzerland) was employed to transfected NK92 cells with 200 nM siRNAs.

### RT-PCR and RT-qPCR

Trizol reagent (Invitrogen) was utilized for the extraction of total RNA and the cDNA Synthesis Kit (11123ES10, Yeasen) was then used to synthesize cDNA RT-PCR was performed with a Bio-Rad Thermal Cycler utilizing the 2 × Hieff™ PCR Master Mix (with dye) (10102ES08, Yeasen). RT-qPCR analysis was conducted with 2 × RealStar Green Fast Mixture with ROX (A303, Genstar) using a Bio-Rad Thermal Cycler following the manufacturer’s protocols. The primer sequences can be found in Supplementary Table 2.

### Western blot

Proteins were extracted utilizing RIPA lysis buffer (P0013B, Beyotime) and quantified using a BCA protein assay kit (23225, Pierce). Equal protein quantities underwent resolution via SDS-PAGE prior to Western blot analysis utilizing enhanced chemiluminescence (Pierce) and ChemiScope (CLiNX). The protein band intensity was assessed utilizing Image J software. The protein expression levels were normalized to β-actin as a reference protein. Antibodies used in the study were anti-NKG2D (1:1000, SAB2500697, Sigma-Aldrich, RRID: AB_10604131), anti-CTCF (1:2500, Ab128873, Abcam, RRID: AB_11144295), anti-SRSF1 (1:1000, Ab38017, Abcam, RRID: AB_882519), anti-RAB5 (1:1000, 2143 T, Cell Signaling Technology, RRID: AB_823625), anti-EEA1 (1:10,000, Ab109110, Abcam, RRID: AB_10863524), anti-RAB7 (1:2000, 55469-1-AP, Proteintech, RRID: AB_11182831), anti-LAMP1 (1:1000, 9091 T, Cell Signaling Technology, RRID: AB_2687579), and anti-β-Actin (1:20000; 66009-1-Ig, Proteintech, RRID: AB_2687938).

### Coimmunoprecipitation (co-IP)

NK92 cells were subjected to lysis at 4 °C using a lysis buffer containing 20 mM Tris–HCl, pH 7.5, 0.5% NP-40, 250 mM NaCl, 3 mM EDTA, and 3 mM EGTA. Following centrifugation, the obtained supernatant underwent overnight immunoprecipitation at 4 °C using suitable antibodies and IgG. Next, the lysate was incubated with Pierce protein A/G Magnetic Beads (88803, Thermo Fisher Scientific), washed multiple times with the lysis buffer, and then evaluated via Western blotting analysis. The antibodies used were anti-CTCF (Ab128873, Abcam, RRID: AB_11144295), anti-EZH2 (21800-1-AP, Proteintech, RRID: AB_10858790), anti-SUZ12 (20366-1-AP, Proteintech, RRID: AB_10694152), and anti-GAPDH (60004-1-Ig, Proteintech, RRID: AB_2107436).

### Flow cytometry

For surface staining, both NK92 and human primary activated NK cells underwent incubation with APC-anti-human NKG2D antibody (320808, Biolegend, RRID: AB_492962), APC-anti-human TIGIT antibody (372706, Biolegend, RRID: AB_2632732), and APC-anti-human CD96 antibody (338409, Biolegend, RRID: AB_2566141) at 4 °C for 20 min in 1xPBS buffer. For intracellular staining, cytokine staining was performed on NK92 and human primary activated NK cells utilizing the BD Cytofix/Cytoperm Fixation/Permeabilization Solution Kit with BD GolgiPlug (555028, BD Biosciences) with PE-anti-human IFN-γ antibody (502509, Biolegend, RRID: AB_315234), APC-anti-human CD107a antibody (560664, BD Biosciences, RRID: AB_1727417), and PE-anti-human/mouse Granzyme B recombinant antibody (372208, Biolegend, RRID: AB_2687032). Following staining, the cells underwent buffer washing before being analyzed with a BD FACS Aria II flow cytometer (BD Biosciences). The acquired data underwent analysis utilizing version 10 of FlowJo software (Tree Star, Ashland, OR, USA).

### Immunofluorescence

NK92 or human primary activated NK cells were subjected to sequential centrifugation and PBS washing, followed by fixation at room temperature with 4% paraformaldehyde for 10 min. Subsequently, permeabilization was performed using 0.5% Triton X100 for 10 min at 4 °C. Finally, the cells were centrifuged and dried on slides. After the aforementioned steps, the cells were further treated with 1% BSA in PBS for blocking and subsequently incubated with antibodies against NKG2D (DF4816, Affinity Biosciences, RRID: AB_2837167), *RAB5A* (46449 T, Cell Signaling Technology, RRID: AB_2799303), and CTCF (Ab128873, Abcam, RRID: AB_11144295). Following the overnight incubation at 4 °C, the cells underwent incubation with fluorescent secondary antibodies conjugated with Alexa Fluor 555 (A-31570, Invitrogen, RRID: AB_2536180) or Alexa Fluor 488 (A-11008, Invitrogen, RRID: AB_143165) for 1 h at room temperature. The cells were then stained with 1 × DAPI at room temperature for 10 min, washed with PBS and fixed on slides. Images were acquired with an FV3000 confocal microscope (Olympus, Tokyo, Japan).

### *Soluble MICA*-treatment assay

NK92 or human primary activated NK cells were seeded into 24-well plates with 1 × 10^5^ cells per well and subsequently treated with varying concentrations (0, 100, 200, 400, 800, 1000, 2000, 3000, and 4000 pg/ml) of rsMICA protein (MIA-H5221, Acro Biosystems) at 37 °C for 24 h. Subsequently, cells were harvested and processed for different experiments.

### Actinomycin D assay

NK92 cells were cultured in 24-well plates with 1 × 105 cells per well and subsequently treated with 2 μg/ml actinomycin D (HY-17559, MedChemExpress). At various time intervals (0, 4, 8, 16, and 24 h) after actinomycin D treatment, cells were harvested. RT-qPCR was performed to analyze the expression of circular and linear *ARAP2*, with normalization of the values to those in the 0-h group.

### RNase R assay

NK92 cell-derived total RNA was subjected to incubation with RNase R (R7092S, Beyotime) at 37 °C for 10 min. Subsequently, RT-qPCR was conducted to verify the durability of circular and linear *ARAP2* to RNase R digestion.

### *RNA fluorescence *in situ* hybridization (FISH)*

The Cy3-labelled FISH probe that spans the back-splicing junction of *circARAP2* was obtained from RiboBio (Guangzhou, China). The Cy3-labelled FISH probes for nuclear reference U6 and cytoplasmic reference 18S were also provided by RiboBio. The FISH assay was conducted using the Ribo™ Fluorescent in Situ Hybridization Kit (Ribo). Image acquisition was carried out with an FV3000 confocal microscope (Olympus, Tokyo, Japan).

### Cytoplasm-nucleus separation assay

For investigating the subcellular localization of *circARAP2*, we utilized the PARIS™ kit (AM1921, Invitrogen, Thermo Fisher Scientific, Inc.) to achieve the isolation of the cytoplasmic and nuclear RNA fractions. qRT-PCR was carried out to assess the expression of cytoplasmic control ACTIN, nuclear control U2, and *circARAP2* in both cytoplasmic and nuclear fractions of rsMICA-treated and untreated NK92 cells.

### Calcein AM cytotoxicity assay

Labeled with Calcein-AM obtained from Dojindo Laboratories, 100 μL of K562 cells (target cell) were seeded into 96-well plates with 5 × 103 cells per well. Subsequently, 100 μL of lentiviruses transfected NK92 cells (effector cell), with or without rsMICA treatment, were added to each well at different effector-to-target cell ratios. The target cells were incubated in medium alone to achieve minimum release, while treatment with 0.4% Triton X-100 promoted maximum release. Incubation of the plates was performed at 37 °C. Following a 4-h incubation period, the supernatant (100 μL) from each well was carefully transferred into new 96-well plates. Subsequently, a Synergy HT Microplate Reader (BioTek Instruments, Winooski, VT, USA) was utilized to acquire the results.

### Enzyme-linked immunosorbent assay (ELISA)

We seeded 3 × 106 K562 cells in each well of a 24-well plate and collected the supernatants at 24, 48, 72, 96, and 120 h for ELISA detection. ELISA was conducted using the Human MICA ELISA kit (ab100592, Abcam) to obtain the quantitative measurement of soluble MICA in K562 cell culture supernatants.

### Animal studies

Immunodeficient female NCG mice, aged 6–8 weeks, were acquired from Nanjing Biomedical Research Institute of Nanjing University and subsequently kept in an SPF barrier environment. The mice were randomly assigned to four groups. On day 0, the mock group was administered 3 × 106 K562 cells labeled with luciferase via the tail vein. Meanwhile, the shCtrl, sh*circARAP2*_1, and sh*circARAP2*_2 groups were each administered 3 × 106 luciferase-labeled K562 cells and 3 × 106 transfected NK92 cells via the tail vein. On day 8 and day 15, an equivalent amount of transfected NK92 cells was administered via the tail vein. Bioluminescence imaging was carried out on days 7, 14, and 21 by intraperitoneal delivery of D-luciferin (LUCK-1G, Goldbio). All mice were administered 5000 IU IL-2 via the intraperitoneal route every other day for 21 days. All mice were routinely monitored, and their deaths were recorded to create a survival curve.

### Chromatin isolation by RNA Purification (ChIRP) assay

The ChIRP assay was conducted utilizing the Chromatin isolation by RNA purification (ChIRP) Kit (Bes5104, BersinBio, China). ChIRP probes targeting the back-splicing junction of *circARAP2* were provided by RiboBio. The ChIRP probe sequences are provided as follows: 5′-AATAGCCTTGAGGAGAGAGT-/3bio/-3′ and 5′-TTTCACGGAAATAGCCTTGA-/3bio/-3′. The obtained DNA from ChIRP was then sequenced to identify possible target genes that may interact with *circARAP2*.

### RNA pull-down assay

The RNA pull-down assay was conducted using the Pierce™ Magnetic RNA–Protein Pull-down Kit (20164, Thermo Scientific). *CircARAP2* was transcribed from DNA templates utilizing the HiScribe™ T7 Quick High Yield RNA Synthesis Kit (E2050S, NEB) and subsequently labelled utilizing the Pierce™ RNA 3' End Desthiobiotinylation Kit (20163, Thermo Scientific) to synthesize the biotin-labeled *circARAP2* probe. The proteins extracted after the RNA pull-down assay were separated using SDS-PAGE and visualized using silver staining (36244ES30, Yeasen). Subsequently, mass spectrometry analysis was conducted on the proteins. The identity of the CTCF protein was further confirmed by Western blotting utilizing an anti-CTCF antibody (Ab128873, Abcam, RRID: AB_11144295).

### RNA immunoprecipitation (RIP) assay

RIP assays were conducted employing an RNA Immunoprecipitation Kit (P0101, GENESEED), following the manufacturer's protocols. Antibodies that are specific to CTCF (Ab128873, Abcam, RRID: AB_11144295) and SRSF1 (Ab38017, Abcam, RRID: AB_882519), respectively, were utilized to capture the endogenous complex of protein and RNA. A negative control was included using normal rabbit IgG. Following the purification of RNAs, specific primers targeting *circARAP2* or pre-*ARAP2* were utilized for reverse transcription and qPCR. The primer sequences can be found in Supplementary Table 2.

### Luciferase reporter assay

To access the transcriptional activity of *RAB5A* promoter, the 2000 bp promoter region of *RAB5A* was inserted into the pGL3 basic firefly luciferase reporter vector provided by Promega. The constructed pGL3-*RAB5A* plasmids were co-transfected with CTCF overexpression or knockdown plasmids and pRL-TK Renilla luciferase Reporter Vector (Promega) plasmids into 293 T cells. Cellular lysates were collected after 48 h of transfection, and the firefly and Renilla luciferase activities were evaluated utilizing the Dual-Luciferase Reporter Assay System (E1910, Promega), following the recommended protocols provided by the manufacturer. The pRL-TK Renilla luciferase Reporter Vector was used for data normalization.

### RNA EMSA

For RNA EMSA, biotin-labeled RNA probes for 376–427 bp of *circARAP2* were in vitro transcribed and subsequently labelled as mentioned before. The full-length CTCF recombinant protein was obtained from OriGene. RNA EMSA was conducted following the manufacturer's protocols of LightShift Chemilumyinescent RNA EMSA Kit (20158, Thermo Fisher Scientific). Antibodies specific to CTCF (Ab128873, Abcam, RRID: AB_11144295) were used to identify the combination between CTCF and the probes.

### Chromatin immunoprecipitation (ChIP) assay

ChIP assays were carried out utilizing a Pierce Magnetic ChIP Kit (26157, Thermo Fisher Scientific), following the guidelines provided by the manufacturer. Antibodies specific to CTCF (Ab128873, Abcam, RRID: AB_11144295), trimethyl-H3K9 (39062, Active Motif, RRID: AB_2532132), and trimethyl-H3K27 (39055, Active Motif, RRID: AB_2561020) were employed to investigate the promoter profile of *RAB5A*. A negative control was included using normal rabbit IgG. Purified DNAs were subjected to qPCR with specific primers designed to target the *RAB5A* promoter region, and the primer sequences can be found in Supplementary Table 2.

### In vitro triplex formation assay

The in vitro triplex formation assay was adapted from previous studies [61, 62]. In this assay, Cy3-labeled DNA probes and Cy5-labeled RNA probes were incubated together in a triplex-binding buffer at room temperature for at least 2 h. The buffer consists of 90 mM Tris–acetate pH 6.5, 10 mM MgCl_2_, 100 µg/mL tRNA and 0.5 mM spermine tetrahydrochloride. Triplex formation was then assessed by conducting electrophoresis on 8% native polyacrylamide gels and visualized using Sapphire™ Biomolecular Imager.

### Statistical analysis

The statistical significance of the data was determined utilizing the GraphPad Prism version 8 software, employing either the paired/unpaired two-tailed Student's t-test or one/two-way ANOVA. Data are expressed as mean ± SEM. The level of significance among groups is depicted in the following manner: ns: no significance, *P < 0.05, **P < 0.01, ***P < 0.001, ****P < 0.0001.

### Supplementary Information

Below is the link to the electronic supplementary material.

Supplementary file1 (PDF 169 KB)

Supplementary file2 (PDF 235 KB)

Supplementary file3 (PDF 12216 KB)

Supplementary file4 (DOC 17 KB)

## Data Availability

Data are available on reasonable request.
