# Supplementary material for: CircARAP2 controls sMICA-induced NK cell desensitization by erasing CTCF/PRC2-induced suppression in early endosome marker RAB5A
Source: Cell Mol Life Sci. 2024 Jul 24;81(1):307. doi: 10.1007/s00018-024-05285-1 (PMC11335232; doi:10.1007/s00018-024-05285-1)
Supplement: Supplementary file 1 — Supplementary file1 (PDF 169 KB) [file 18_2024_5285_MOESM1_ESM.pdf]

CircARAP2* controls MICA-induced NK cell desensitization by erasing CTCF/PRC2-induced suppression in early endosome marker *RAB5A

Cellular and Molecular Life Sciences

Feifei Guo[1], Nawen Du[1], Xue Wen[1], Zhaozhi Li[1], Yantong Guo[1], Lei Zhou[1], Andrew R. Hoffman[2], Lingyu Li[1#], Ji-Fan Hu[1,2#], Jiuwei Cui[1#]

[1] Cancer Center, The First Hospital of Jilin University, Changchun, 130021, China

[2] Stanford University School of Medicine, VA Palo Alto Health Care System, Palo Alto, CA 94304, USA

Correspondence to: Jiuwei Cui, M.D., Ph.D., Cancer Center, The First Hospital of Jilin University, 71 Xinmin Street, Changchun 130021, China, Tel: 86-43188782178, Fax: 86-43188786134, e-mail: cuijw@jlu.edu.cn; Ji-Fan Hu, M.D., Ph.D., Department of Medicine, PAVIR, VA Palo Alto Health Care System, Palo Alto, CA 94304, USA, Tel: 650-852-3275, Fax: 650-856-8024, e-mail: jifan@stanford.edu; jifanhu@jlu.edu.cn; Lingyu Li, M.D., Ph.D., Cancer Center, The First Hospital of Jilin University, 71 Xinmin Street, Changchun 130021, China, e-mail: lilingyu@jlu.edu.cn.

Supplementary Table S1 A list of siRNA sequences used in this manuscript

Target gene	Primers	Sequence	Name
CTCF	Sense	5'-GCCUGCCGUAGAAAUUGAATT-3'	CTCF-homo-
	Antisense	5'-UUCAAUUUCUACGGCAGGCTT-3'	2325
	Sense	5'-GGCAAGACAUGCUGAUAAUTT-3'	CTCF-homo-
	Antisense	5'-AUUAUCAGCAUGUCUUGCCTT-3'	2154
RAB5A	Sense	5'-GUCCUAUGCAGAUGACAAUTT-3'	RAB5A-homo-
	Antisense	5'-AUUGUCAUCUGCAUAGGACTT-3'	980
	Sense	5'-CCAGUUCAAACUAGUACUUTT-3'	RAB5A-homo-
	Antisense	5'-AAGUACUAGUUUGAACUGGTT-3'	592
SRSF1	Sense	5'-GCCCAGAAGUCCAAGUUAUTT-3'	SRSF1-homo-
	Antisense	5'-AUAACUUGGACUUCUGGGCTT-3'	797
	Sense	5'-GGAACAACGAUUGCCGCAUTT-3'	SRSF1-homo-
	Antisense	5'-AUGCGGCAAUCGUUGUUCCTT-3'	244
NC	Sense	5'-UUCUCCGAACGUGUCACGUTT-3'	NC
	Antisense	5'-ACGUGACACGUUCGGAGAATT-3'	
