# Supplementary material for: CircARAP2 controls sMICA-induced NK cell desensitization by erasing CTCF/PRC2-induced suppression in early endosome marker RAB5A
Source: Cell Mol Life Sci. 2024 Jul 24;81(1):307. doi: 10.1007/s00018-024-05285-1 (PMC11335232; doi:10.1007/s00018-024-05285-1)
Supplement: Supplementary file 2 — Supplementary file2 (PDF 235 KB) [file 18_2024_5285_MOESM2_ESM.pdf]

CircARAP2 controls MICA-induced NK cell desensitization by erasing CTCF/PRC2-induced suppression in early endosome marker _RAB5A_

Cellular and Molecular Life Sciences

Feifei Guo[1], Nawen Du[1], Xue Wen[1], Zhaozhi Li[1], Yantong Guo[1], Lei Zhou[1], Andrew R. Hoffman[2], Lingyu Li[1#], Ji-Fan Hu[1,2#], Jiuwei Cui[1#]

[1] Cancer Center, The First Hospital of Jilin University, Changchun, 130021, China

[2] Stanford University School of Medicine, VA Palo Alto Health Care System, Palo Alto, CA 94304, USA

Correspondence to: Jiuwei Cui, M.D., Ph.D., Cancer Center, The First Hospital of Jilin University, 71 Xinmin Street, Changchun 130021, China, Tel: 86-43188782178, Fax: 86-43188786134, e-mail: cuijw@jlu.edu.cn; Ji-Fan Hu, M.D., Ph.D., Department of Medicine, PAVIR, VA Palo Alto Health Care System, Palo Alto, CA 94304, USA, Tel: 650-852-3275, Fax: 650-856-8024, e-mail: jifan@stanford.edu; jifanhu@jlu.edu.cn; Lingyu Li, M.D., Ph.D., Cancer Center, The First Hospital of Jilin University, 71 Xinmin Street, Changchun 130021, China, e-mail: lilingyu@jlu.edu.cn.

Supplementary Table S2 Primer sets used in the manuscript

RT-PCR and RT-qPCR

Primer set	Primers	Sequence	Product size (bp)
β-Actin	Forward	5'-CAGGTCATCACCATTGGCAATGAGC-3'	134
	Reverse	5'-CGGATGTCCACGTCACACTTCATGA-3'	
ARAP2	Forward	5'-GCATGAGGGGAATGCAACCT-3'	181
	Reverse	5'-TCGGGGTCGATTTCGAAGTTT-3'	
CircARAP2	Forward	5'-TGGACTCCGTTAATAGGCACA-3'	230
	Reverse	5'-CTCATTTGAATTTTGCCATGCCA-3'	
NKG2D	Forward	5'-CAAGATCTTCCCTCTCTGAGCA-3'	105
	Reverse	5'-CCACGAATCCACCCCATCAA-3'	
U2	Forward	5'-ATCTGTTCTTATCAGTTTAATATCTG-3'	151
	Reverse	5'-GGGTGCACCGTTCCTGGAGGTAC-3'	
SRSF1	Forward	5'-TCAGGCAAGGTTGTCCAAGT-3'	249
	Reverse	5'-GTAACTGCGACTCCTGCTGT-3'	
Convergent	Forward	5'-TGGGCAACCAAAAGGCCATA-3'	147
	Reverse	5'-GGAAGGGTTCTTGACCGTTG-3'	

RIP

Primer set	Primers	Sequence	Product size (bp)
Pre-mRNA	Forward	5'-AGCCGTTTCTTCACAGGCAAT-3'	181
	Reverse	5'-CAGCAACCCTGGAGGTTTAGAA-3'	

ChIP

Primer set	Primers	Sequence	Product size (bp)
RAB5A-set1	Forward	5'-ATGGACCAGGTGTTGGGATTAG-3'	170
	Reverse	5'-ACTCCAAACCTGCTTCTGCTT-3'	
RAB5A-set2	Forward	5'-CCGACTGTACGCCCATAAGA-3'	217
	Reverse	5'-ACATTCAGTCTCTGGCGAGG-3'	
RAB5A-set3	Forward	5'-TTTACAGCGGTGAGGACCAC-3'	196
	Reverse	5'-CGGGGAAGGCCAAATCCTAA-3'	
RAB5A-set4	Forward	5'-TAACAATGGCGCTGGAGAGG-3'	205
	Reverse	5'-TCGTCACTTCCCATCATCGC-3'	

RNA pull-down

Primer set	Primers	Sequence
Sense	Forward	5'-TAATACGACTCACTATAGGGCTATTTCCGTGAAAG-3'
	Reverse	5'-CCTTGAGGAGAGAGTTTATCCA-3'
Anti-sense	Forward	5'-TAATACGACTCACTATAGGGCCTTGAGGAGAGAGTTTAT-3'
	Reverse	5'-CTATTTCCGTGAAAGAAGAAA-3'

EMSA

Primer set	Primers	Sequence
Binding-sequence	Forward	5'-TAATACGACTCACTATAGGG-3'
	Reverse	5'-GTCCAAAATCAAGGCTTTTT-3'
