# Supplementary material for: CircARAP2 controls sMICA-induced NK cell desensitization by erasing CTCF/PRC2-induced suppression in early endosome marker RAB5A
Source: Cell Mol Life Sci. 2024 Jul 24;81(1):307. doi: 10.1007/s00018-024-05285-1 (PMC11335232; doi:10.1007/s00018-024-05285-1)
Supplement: Supplementary file 3 — Supplementary file3 (PDF 12216 KB) [file 18_2024_5285_MOESM3_ESM.pdf]

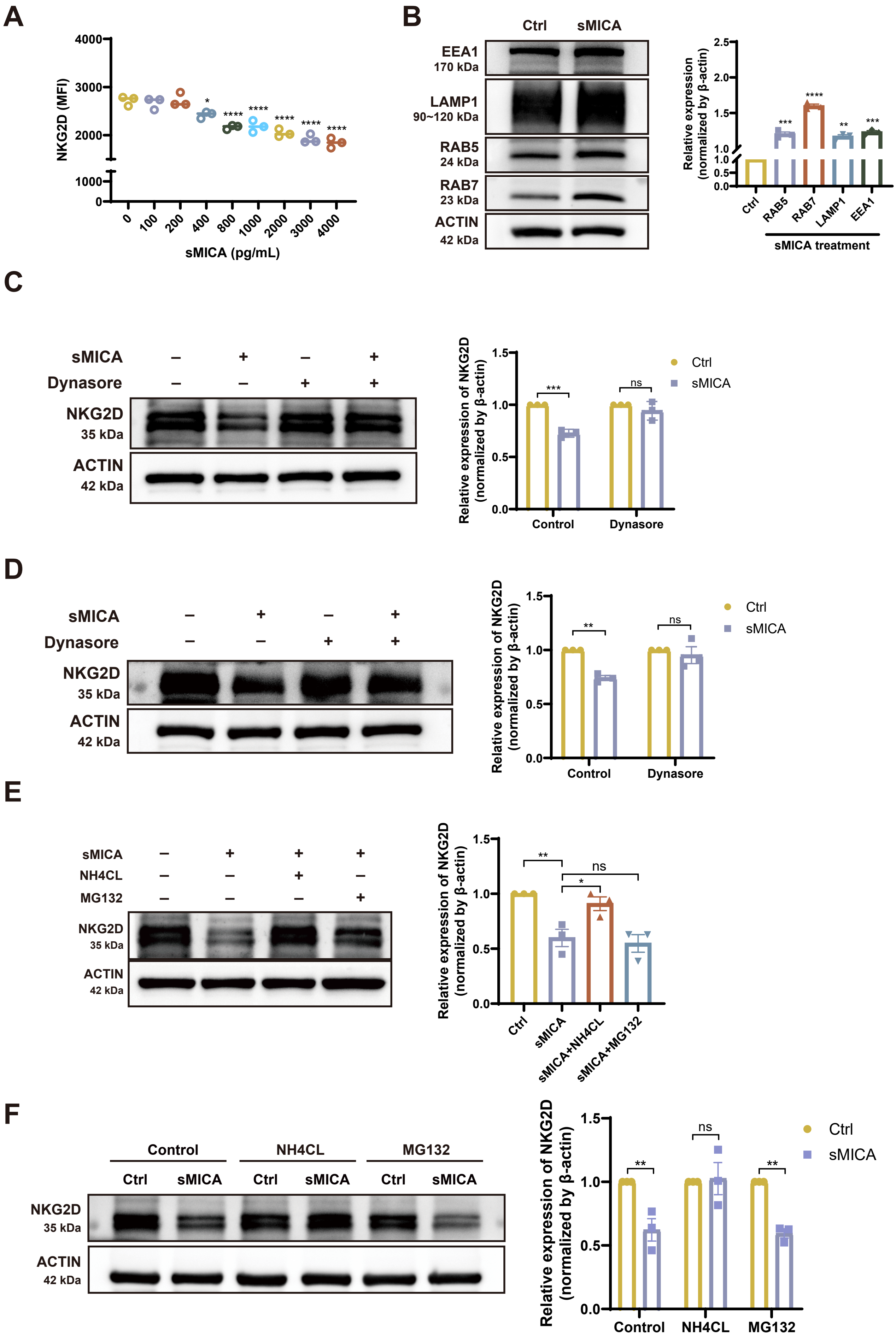

Figure S1. CircARAP2 in NK cells

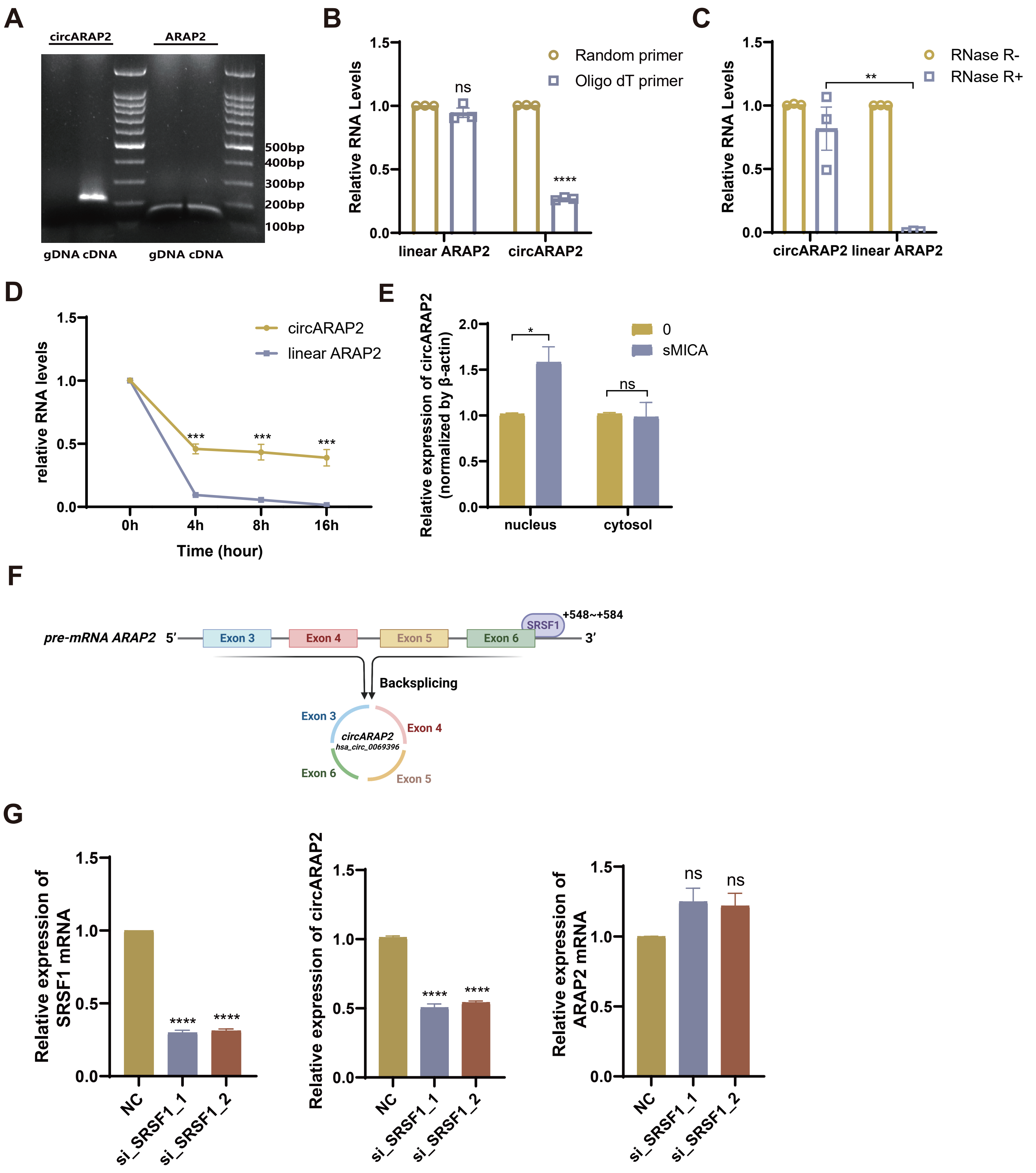

Figure S2. Circular characteristics of CircARAP2

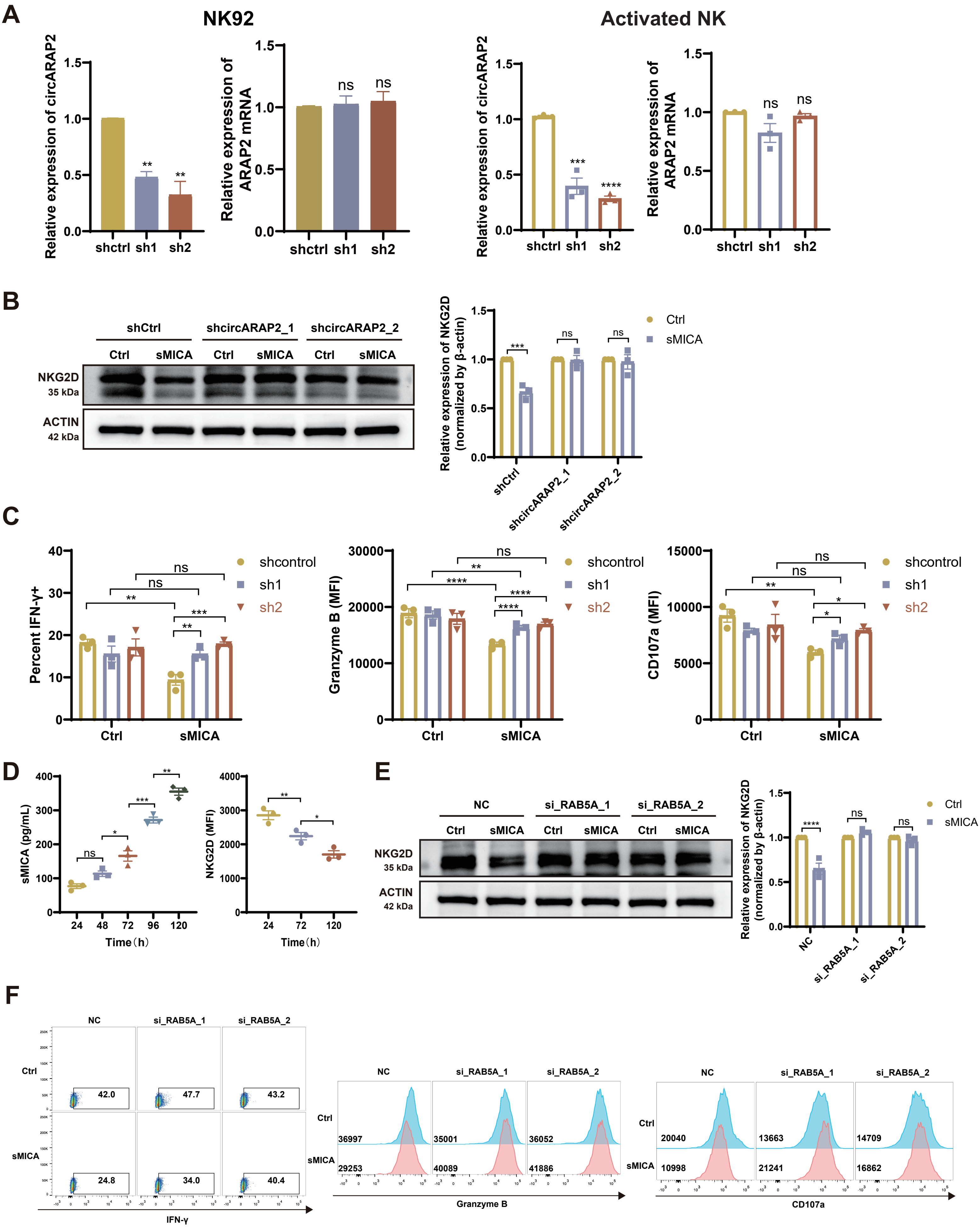

Figure S3. CircARAP2 affects IFN-γ, Granzyme B and CD107a expression in NK cells

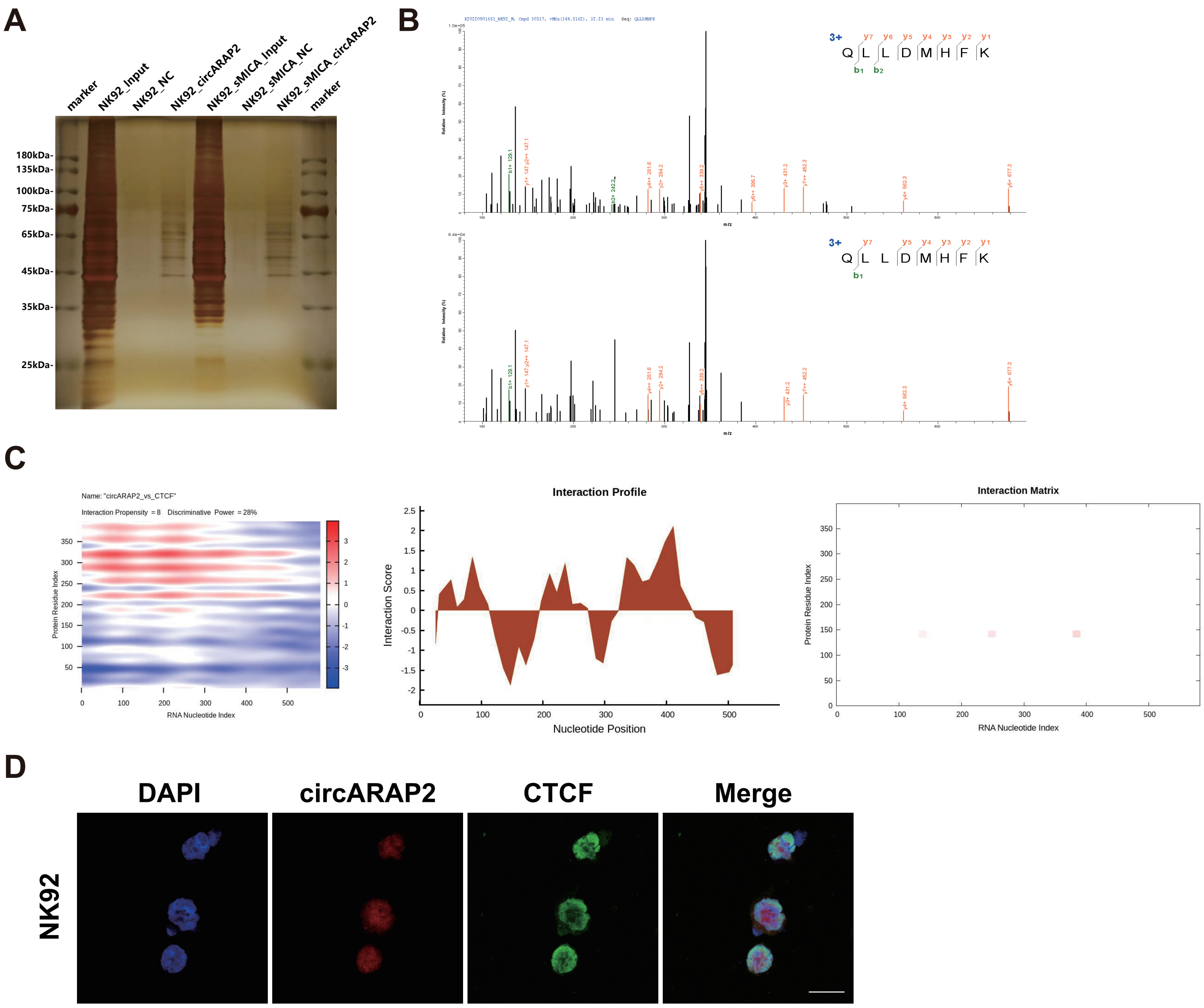

Figure S4. Identificaiton of CircARAP2-interacting factors

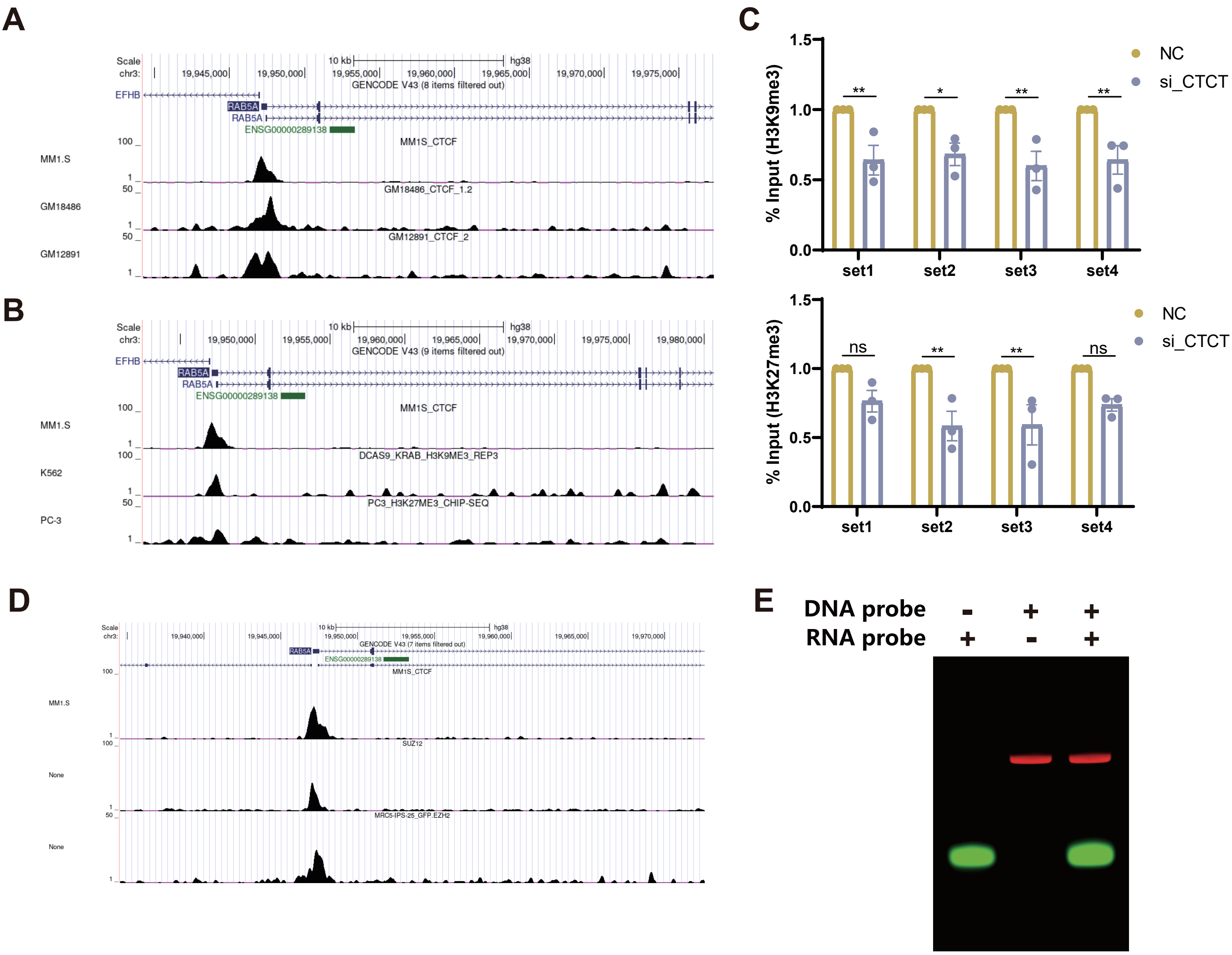

Figure S5. CTCF regulates histone methylation in the RAB5A promoter
