# Supplementary material for: CircARAP2 controls sMICA-induced NK cell desensitization by erasing CTCF/PRC2-induced suppression in early endosome marker RAB5A
Source: Cell Mol Life Sci. 2024 Jul 24;81(1):307. doi: 10.1007/s00018-024-05285-1 (PMC11335232; doi:10.1007/s00018-024-05285-1)
Supplement: Supplementary file 4 — Supplementary file4 (DOC 17 KB) [file 18_2024_5285_MOESM4_ESM.docx]

**Supplmental figure legend**

**Fig. S1 *CircARAP2* in NK cells**

**(A)** Flow cytometry analysis of NKG2D expression on NK92 cells following a 24-hour treatment with varying concentrations (0, 100, 200, 400, 800, 1000, 2000, 3000, and 4000 pg/ml) of rsMICA. **(B)** Immunoblot of endocytic marker proteins in NK92 cells treated with rsMICA. **(C)** NKG2D in rsMICA-treated NK92 cells. NK92 cells were preincubated for 30 min in medium containing 100 μM dynasore or dimethyl sulfoxide (DMSO) at 37°C. The cells then underwent rsMICA treatment and analysis of western blot. **(D)** Primary activated NK cells undergo the same treatment and analysis as in Fig. S1C. **(E)** NK92 cells were preincubated overnight in medium containing 20 mM NH_4_CL or for 4 hours in medium containing 10 μM MG132. Afterwards, the cells were subjected to rsMICA treatment followed by western blot analysis. **(F)** Primary activated NK cells undergo the same treatment and analysis as in Fig. S1E. For A, B, E, data are presented of three independent experiments, one-way ANOVA. For C, D, F, data are presented of three independent experiments, two-way ANOVA. (ns: no significance, *P < 0.05, **P < 0.01, ***P < 0.001, ****P < 0.0001).

**Fig. S2 Circular characteristics of *circARAP2***

**(A)** For PCR amplification of *circARAP2* and linear *ARAP2*, divergent and convergent primers were employed. **(B)** RNA extracted from NK92 cells was reverse transcribed with oligo(dT) primers or random primers. *CircARAP2* or linear *ARAP2* mRNA was detected by qPCR. **(C)** Relative levels of *circARAP2* and linear *ARAP2* after treatment with RNase R. **(D)** Half-lives of *circARAP2* and linear *ARAP2* mRNA after treatment with actinomycin D (2 μg/ml). **(E)** The cytoplasmic and nuclear levels of circ*ARAP2* in both rsMICA-treated and untreated activated NK cells. **(F)** The schematic diagram showed the predicted binding site of SRSF1 on pre-*ARAP2*. **(G)** After transfecting with siRNA targeting SRSF1 and control, *circARAP2* and linear *ARAP2* expression level was assessed by RT-qPCR. si_SRSF1_1 and _2 represent two different sets of siRNA, data are presented of three independent experiments, one-way ANOVA. For B, C, D, E, data are presented of three independent experiments, two-way ANOVA. (ns: no significance, *P < 0.05, **P < 0.01, ***P < 0.001, ****P < 0.0001).

**Fig. S3 CircARAP2 a­ffects IFN-γ, Granzyme B and CD107a expression in NK cells**

**(A)** The RT-qPCR analysis was conducted to verify the relative expression levels of *circARAP2* and linear *ARAP2* in NK92 and primary activated NK cells that were stably transfected with scramble shRNA (shCtrl) and shcric*ARAP2*. **(B)** Immunoblot of NKG2D in NK92 cells stably transfected with shCtrl and shcric*ARAP2*, with or without rsMICA treatment. **(C)** IFN-γ, Granzyme B and CD107a expression was measured in primary activated NK cells transfected with shCtrl or shcric*ARAP2*, with or without rsMICA treatment. **(D)** Left panel, The concentration of soluble MICA in the supernatant of K562 cells at different culture times was measured using ELISA; Right panel, Flow cytometry analysis for NKG2D expression on NK92 cells treated with supernatant of K562 cells collected at 24, 72, and 120 hours. **(E)** Immunoblot of NKG2D in NK92 cells transfected with siRNA targeting *RAB5A* and control, with or without rsMICA treatment. **(F)** IFN-γ, Granzyme B and CD107a expression was measured in NK92 cells transfected with siRNA targeting *RAB5A* and control, with or without rsMICA treatment. For A and D, data are presented of three independent experiments, one-way ANOVA. For B, C, E, data are presented of three independent experiments, two-way ANOVA. (ns: no significance, *P < 0.05, **P < 0.01, ***P < 0.001, ****P < 0.0001).

**Fig. S4 Identification of *CircARAP2*-interacting factors**

**(A)** Silver staining for protein products from RNA pull-down assays using *circARAP2* specific probes. **(B)** The peak map of CTCF acquired from the RNA pull-down mass spectrometry assay. **(C)** The data from catRAPID platform showing potential interaction propensity and binding sites between CTCF and *circARAP2*. **(D)** Combined FISH and IF was conducted utilizing a probe against *circARAP2* and anti-CTCF antibody (Scale bar, 20μm).

**Fig. S5 CTCF regulates histone methylation in the RAB5A promoter**

**(A)** The recruitment of CTCF on the promoter region of *RAB5A* can be observed on the UCSC Genome Browser. **(B)** The recruitment of H3K9me3 and H3K27me3 surrounding CTCF binding sites on the *RAB5A* promoter can be observed on the UCSC Genome Browser. **(C)** ChIP and RT-qPCR assays demonstrate alterations in the association of H3K9me3 and H3K27me3 with the *RAB5A* promoter in NK92 cells following transfection with CTCF-targeting siRNA and a control. Data are presented of three independent experiments, two-way ANOVA. **(D)** The recruitment of EZH2 and SUZ12 surrounding CTCF binding sites on the promoter region of *RAB5A* can be observed on the UCSC Genome Browser. **(E)** The results of the in vitro triplex formation assay suggest that there was no detectable interaction between the RNA and DNA probes. (ns: no significance, *P < 0.05, **P < 0.01).
